# Estimating economic and disease burden of snakebite in ASEAN countries using a decision analytic model

**DOI:** 10.1371/journal.pntd.0010775

**Published:** 2022-09-28

**Authors:** Chanthawat Patikorn, Jörg Blessmann, Myat Thet Nwe, Patrick Joseph G. Tiglao, Taksa Vasaruchapong, Tri Maharani, Uyen Vy Doan, Syafiq Asnawi Zainal Abidin, Ahmad Khaldun Ismail, Iekhsan Othman, Suthira Taychakhoonavudh, Nathorn Chaiyakunapruk

**Affiliations:** 1 Department of Social and Administrative Pharmacy, Faculty of Pharmaceutical Sciences, Chulalongkorn University, Bangkok, Thailand; 2 Department of Implementation Research, Bernhard Nocht Institute for Tropical Medicine, Hamburg, Germany; 3 Myanmar Snakebite Project, Mandalay, Myanmar; 4 Department of Emergency Medicine, Eastern Visayas Regional Medical Center, Tacloban City, Philippines; 5 Philippine Toxinology Society, Incorporated, Manila, Philippines; 6 Department of Emergency Medicine, University of the Philippines-Manila, Philippine General Hospital, Manila, Philippines; 7 Department of Emergency Medicine, Corazon Locsin Montelibano Memorial Regional Hospital, Bacolod City, Negros Occidental, Philippines; 8 Snake Farm, Queen Saovabha Memorial Institute, The Thai Red Cross Society, Bangkok, Thailand; 9 National Institute Research and Development, Ministry of Health, Jakarta, Indonesia; 10 Division of Medical Toxicology, Cho Ray Hospital, Ho Chi Minh City, Vietnam; 11 Jeffrey Cheah School of Medicine and Health Sciences, Monash University Malaysia, Bandar Sunway, Selangor, Malaysia; 12 Department of Emergency Medicine, Faculty of Medicine, Universiti Kebangsaan Malaysia, Bandar Tun Razak, Kuala Lumpur, Malaysia; 13 Department of Pharmacotherapy, College of Pharmacy, University of Utah, Salt Lake City, Utah, United States of America; 14 IDEAS Center, Veterans Affairs Salt Lake City Healthcare System, Salt Lake City, Utah, United States of America; 15 School of Pharmacy, Monash University Malaysia, Selangor, Malaysia; University of Peradeniya Faculty of Medicine, SRI LANKA

## Abstract

**Background:**

Understanding the burden of snakebite is crucial for developing evidence-informed strategies to pursue the goal set by the World Health Organization to halve morbidity and mortality of snakebite by 2030. However, there was no such information in the Association of Southeast Asian Nations (ASEAN) countries.

**Methodology:**

A decision analytic model was developed to estimate annual burden of snakebite in seven countries, including Malaysia, Thailand, Indonesia, Philippines, Vietnam, Lao PDR, and Myanmar. Country-specific input parameters were sought from published literature, country’s Ministry of Health, local data, and expert opinion. Economic burden was estimated from the societal perspective. Costs were expressed in 2019 US Dollars (USD). Disease burden was estimated as disability-adjusted life years (DALYs). Probabilistic sensitivity analysis was performed to estimate a 95% credible interval (CrI).

**Principal findings:**

We estimated that annually there were 242,648 snakebite victims (95%CrI 209,810–291,023) of which 15,909 (95%CrI 7,592–33,949) were dead and 954 (95%CrI 383–1,797) were amputated. We estimated that 161,835 snakebite victims (69% of victims who were indicated for antivenom treatment) were not treated with antivenom. Annual disease burden of snakebite was estimated at 391,979 DALYs (95%CrI 187,261–836,559 DALYs) with total costs of 2.5 billion USD (95%CrI 1.2–5.4 billion USD) that were equivalent to 0.09% (95%CrI 0.04–0.20%) of the region’s gross domestic product. >95% of the estimated burdens were attributed to premature deaths.

**Conclusion/Significance:**

The estimated high burden of snakebite in ASEAN was demonstrated despite the availability of domestically produced antivenoms. Most burdens were attributed to premature deaths from snakebite envenoming which suggested that the remarkably high burden of snakebite could be averted. We emphasized the importance of funding research to perform a comprehensive data collection on epidemiological and economic burden of snakebite to eventually reveal the true burden of snakebite in ASEAN and inform development of strategies to tackle the problem of snakebite.

## Author summary

In the Association of Southeast Asian Nations (ASEAN) countries, we estimated that annually there were 242,648 snakebite victims of which 15,909 victims were dead and 954 victims were amputated. We estimated that 69% of victims indicated for antivenom treatment were not treated with antivenom. Annual disease burden of snakebite was estimated at 391,979 disability-adjusted life years (DALYs) with estimated total costs of snakebite of 2.5 billion US Dollars which were equivalent to 0.09% of the region’s gross domestic product. Almost all of the estimated economic and disease burdens were related to deaths from snakebite envenoming which suggested that the remarkably high burden of snakebite could actually be averted. We emphasized the importance of funding research to perform a comprehensive data collection on epidemiological and economic burden of snakebite to eventually reveal the true burden of snakebite in ASEAN and inform development of strategies to tackle the problem of snakebite.

## Introduction

Snakebite is a neglected tropical disease that was estimated to affect 5.4 million victims with up to 138,000 deaths around the world [1]. Snakebite envenoming has been recognized by the World Health Organization (WHO) as the highest priority neglected tropical diseases since 2017. The WHO has set its goal to halve the global morbidity and mortality burden of snakebite by 2030 [2, 3].

The Association of Southeast Asian Nations (ASEAN) is an economic union comprising of ten member countries including Brunei Darussalam, Cambodia, Indonesia, Lao PDR, Malaysia, Myanmar, Philippines, Singapore, Thailand, and Vietnam with over 600 million people [4]. ASEAN is one of the tropical regions with disproportionately high incidence of snakebite. Previous estimation of snakebite in 2007 found that approximately 234,000–1,410,000 people were bitten by snake annually resulting in 700–18,000 deaths in eight ASEAN countries, except Brunei Darussalam and Singapore where snakebite rarely occurred and/or exact data were lacking [1].

Our previous study found that there are five domestic antivenom manufacturers in ASEAN, including Thailand, Indonesia, Philippines, Vietnam, and Myanmar. Up to 290,000 vials of antivenoms were annually produced by these manufacturers which could treat approximately 42,000 victims with snakebite envenoming. However, these produced antivenoms were not enough to treat all victims indicated for antivenom treatment. Besides, the total demand of antivenoms in ASEAN was not estimated [5]. This warranted a comprehensive research on burden of snakebite in the region to quantitatively highlight the neglected problem.

Understanding the current economic and disease burden of snakebite is crucial for developing evidence-informed strategies to reduce morbidity and mortality of snakebite victims to pursue the goal set by the WHO [3]. Studies have been conducted to estimate the annual national economic and disease burden of snakebite in regions where snakebites are prevalent such as Africa [6–12]. Nevertheless, there was no such information in ASEAN countries. Thus, we aimed to estimate economic and disease burden of snakebite in ASEAN using a decision analytic modelling approach.

## Methods

### Ethics statement

This study was approved by the Monash University Research Ethics Committee (Project ID: 23246). Written consent was formally obtained from the participants.

### Overall approaches

A decision analytic model was developed to estimate the annual economic and disease burden of snakebite in seven ASEAN countries including Malaysia, Thailand, Indonesia, Philippines, Vietnam, Lao PDR, and Myanmar. These seven countries were selected based on the evidence of documented snakebite in the country and availability of local key informants to gather more insights on the situation of snakebite which were not publicly available. Brunei Darussalam and Singapore were not included because snakebite rarely occurred and/or exact data were lacking [1]. Cambodia was not included due to lack of recently published literature on snakebite and key informants that hindered the proper estimation of the burden of snakebite in Cambodia.

Annual number of snakebite victims in the region were estimated using a decision analytic model which incorporated treatment seeking behavior to include victims who were not treated in healthcare facilities. Economic burden was estimated from the societal perspective to estimate lifetime costs of snakebite victims which occurred from snakebite episode to long-term consequences. To enable comparison of economic burden between countries, all costs of snakebite were presented as annual national total costs for each country in 2019 USD and converted to the percentage of country’s gross domestic product (GDP) in 2019. Disease burden of snakebite was estimated and quantified as disability-adjusted life years (DALYs) lost due to snakebite in one year in each country.

### Decision analytic model

**Fig 1:**
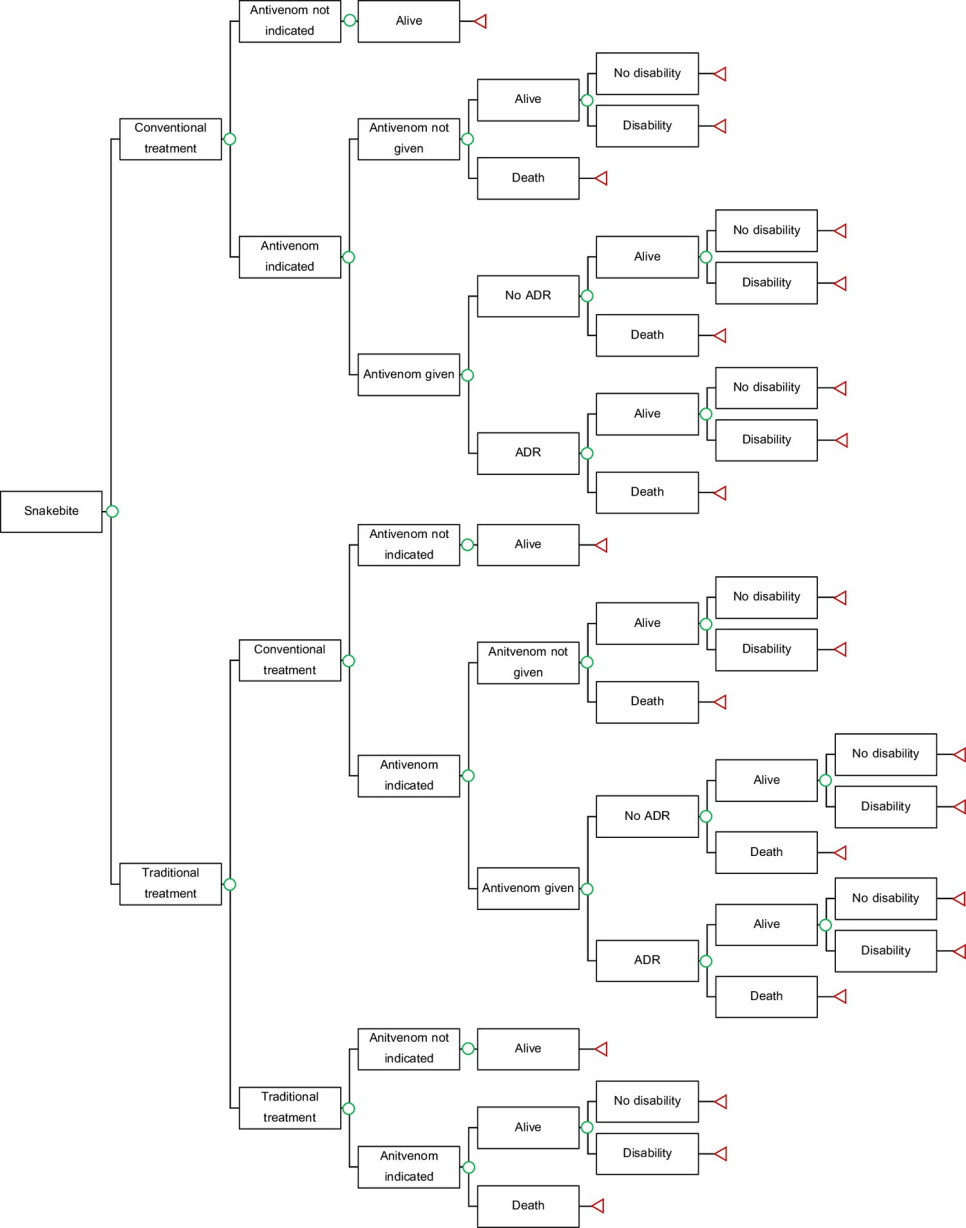
Decision tree to estimate economic and disease burden of snakebite in ASEAN countries. Abbreviation: ADR–adverse drug reaction.

A decision analytic model was developed to simulate the course of snakebite victims in ASEAN which was adapted from previous economic evaluations of antivenoms for snakebite antivenom in West Africa (**Fig 1)** [13, 14]. Victims who were bitten by snake sought for treatment either at conventional treatment (hospitals or healthcare facilities) or traditional treatment through traditional healers to reflect the treatment seeking behavior of victims in the region [5]. Victims who firstly sought traditional healers might subsequently switch to conventional treatment or continue their treatments with traditional healers. Snakebite victims might be indicated for antivenom treatment depending on the occurrence of systemic envenoming following snakebite. Victims who were not indicated for antivenom treatment were assumed to result in being alive as the envenoming is not life-threatening [15–21]. Victims indicated for antivenom treatment who sought conventional treatment might be given with antivenom depending on the current level of access to antivenom in each country. Level of access to antivenom was determined by the number of antivenoms treatment available divided by number of victims indicated for antivenom treatment. Victims who received antivenom treatment might have adverse drug reaction (ADR) following antivenom administration. Victims indicated for antivenom treatment regardless of their treatment seeking behavior might be alive or dead. Alive victims might have disability. Disabilities included in this model were digit and limb amputation.

### Input parameters

Country-specific input parameters were sought from various sources, including published literature, data from the country’s Ministry of Health, local data, and expert opinion (**S1 Table)** [15–62]. An in-depth interview with key informants who were experts in snakebite in ASEAN was also conducted to confirm the retrieved parameters, refer to potential sources of information that might not be publicly available, and ask for their opinions when data were not available. The input parameters were validated through triangulation of data from literature, local data, and interview. Justification of input parameters was described in **S1 Appendix**.

Main sources of information were national statistics and published research for the burden estimation of Malaysia, Thailand, and Myanmar. Published research and anecdotal evidence (local data, and expert opinion) were the main sources of information for the burden estimation of Vietnam, and Lao PDR. Anecdotal evidence was the only source of information for the burden estimation of Indonesia, and Philippines.

### Model assumptions

There were three key assumptions of the model. First, one person can be bitten by snake only once in a lifetime. Second, snakebite victims were accompanied by relatives or family members who took care of them during snakebite episode. Third, antivenom was given to reverse snakebite envenoming and save lives. However, there was no data on the efficacy or effectiveness of antivenom in ASEAN countries. Thus, antivenom effectiveness was based on a study in Nigeria which found a 2.33 fold (95% confidence interval [CI]; 1.26–4.06) increase risk of death in antivenom indicated victims who were not treated with antivenom compared to those treated with antivenom [40]. This relative risk was used to calculate the probability of death due to snakebite in those who were not treated with geographically appropriate antivenoms.

### Total number of snakebite victims

Estimating the total number of snakebite victims occurring in one year in each country was done by applying the country-specific input parameters into the model. The estimated snakebite victims were categorized by their gender, age groups, treatment seeking behavior, indication for antivenom treatment, and disease consequences, i.e., deaths, alive without disabilities, and alive with disabilities.

### Costs of snakebite

Costs of snakebite were estimated from societal perspective, including direct medical costs, direct non-medical costs, and indirect costs (**S1 Table** and **S1 Appendix**). Direct medical costs were estimated using a bottom-up approach which included costs of hospitalization, antivenom treatment, antivenom logistics, ADR management, and amputation. Direct non-medical costs included costs of transportation and additional food for victims and their relatives during snakebite episodes. Indirect costs were estimated using a human capital approach by multiplying the time lost due to illness to the daily income based on the GDP per capita of each country [61]. Indirect costs included productivity losses during snakebite episode of victims and their relatives and productivity losses due to premature death. Productivity losses during snakebite episodes for victims and their relatives were estimated by multiplying length of stay to the daily income. Productivity losses due to premature death were estimated by multiplying the remaining working years from the age of death up to retirement age at 60 years to the GDP per capita. Productivity losses were not quantified for those who died after the age of 60. Productivity losses due to premature death were discounted at the rate of 3% and adjusted for annual growth of GDP per capita in each country [58–60, 62].

### Disease burden of snakebite

Disease burden of snakebite was calculated as DALYs using the template developed by WHO [63]. DALYs were the sum of years of life lost (YLL) and years lived with disability (YLD). YLLs due to snakebite envenoming were calculated from the number of deaths multiplied by a global standard life expectancy at the age of death. YLDs of snakebite victims included YLDs for snakebite episode and YLDs for amputation. YLDs were calculated from the duration of disability multiplied to a disability weight for each condition according to the Global Burden of Disease 2013 study (**S1 Table**) [46].

### Analysis

Economic and disease burden of snakebite in ASEAN was estimated using input parameters as base-case estimates. Sensitivity analyses were performed to assess the model robustness. One-way sensitivity analysis was performed to assess uncertainty of the base-case input parameters over their plausible ranges on the model outputs. Scenario analysis was performed by incorporating post-traumatic stress disorder (PTSD) into the model as a mental disability which estimated that PTSD would occur in 8% (95%CI; 2–18%) of the victims who survived from snakebite envenoming [64]. PTSD could also occur following a snakebite without systemic envenoming. However, the incidence was unknown. Therefore, by applying a lower boundary level of the probability of PTSD following snakebite, it was estimated that 2% of snakebite victims without envenoming would have PTSD following snakebite. Estimation of economic burden of PTSD following snakebite is explained in **S2 Appendix** [65–67]. Probabilistic sensitivity analysis was performed using Monte Carlo simulations for 1,000 times by randomly sampling on a distribution of all parameters to estimate a 95% credible interval (CrI) of the economic and disease burden of snakebite.

### Patient and public involvement

Patients or the public were not involved in the design, or conduct, or reporting, or dissemination plans of our research.

## Results

### Snakebite victims in ASEAN

The model estimated that there were 242,648 snakebite victims (95%CrI 209,810–291,023) annually occurring in ASEAN with annual incidence of 38.03 per 100,000 population (95%CrI 32.89–45.62). The estimated incidence of snakebite ranged from the lowest in Malaysia (10.68 per 100,000 population) to the highest in Lao PDR (200.00 per 100,000 population). (**Tables 1** and **S2**).

**Table 1:** Estimated annual disease burden of snakebite in ASEAN countries.

	Snakebite victims, n	Antivenom indicated victims, n	Deaths, n	Amputations, n	YLLs	YLDs	DALYs	DALYs per 100,000 population
**Malaysia** ^*^	3,412 (3,303–3,533)	481 (254–767)	2 (0–6)	0	50 (0–151)	1.4 (0.6–2.5)	52 (1–152)	0.2 (0.003–0.5)
**Thailand** ^*^	8,715 (8,525–8,906)	5,166 (3,766–6,482)	4 (2–7)	2 (0–7)	102 (51–178)	8 (4–14)	110 (57–185)	0.2 (0.1–0.3)
**Indonesia** ^**+**^	135,000 (134,297–135,689)	49,632 (34,229–65,496)	10,547 (5,012–22,563)	799 (355–1,426)	262,302 (124,650–561,145)	586 (246–1,120)	262,888 (125,252–562,144)	97 (46–208)
**Philippines** ^**+**^	13,377 (11,452–15,772)	1,755 (1,457–2,127)	550 (274–1,099)	12 (6–16)	13,311 (6,624–26,641)	7 (4–11)	13,320 (6,632–26,649)	12 (6–25)
**Vietnam** ^**¶**^	46,745 (17,500–91,013)	41,236 (15,290–80,701)	1,655 (490–4,440)	0	40,136 (11,869–107,679)	114 (38–258)	40,250 (11,931–107,876)	42 (12–112)
**Lao PDR** ^**¶**^	14,339 (14,111–14,571)	3,029 (2,917–3,138)	1,007 (510–2,009)	141 (22–348)	24,468 (12,420–48,837)	61 (10–189)	24,532 (12,462–48,880)	342 (174–682)
**Myanmar** ^*^	21,059 (20,623–21,540)	16,275 (15,877–16,679)	2,145 (1,303–3,824)	0	50,786 (30,877–90,632)	44 (27–67)	50,830 (30,926–90,673)	94 (57–168)
**Total**	242,648 (209,810–291,023)	117,575 (73,790–175,390)	15,909 (7,592–33,949)	954 (383–1,797)	391,154 (186,491–835,263)	825 (329–1,661)	391,979 (187,261–836,559)	61 (29–131)

Estimates are presented as base-case estimates with their 95% credibility interval (in parentheses) based on probabilistic sensitivity analysis. Abbreviations: DALYs–disability-adjusted life years; YLDs–years lived with disabilities; YLLs–years of life lost

* input parameters were based on national statistics and published literature

^¶^ Input parameters were based on published literature and anecdotal evidence

^+^ Input parameters were based on anecdotal evidence.

**Fig 2:**
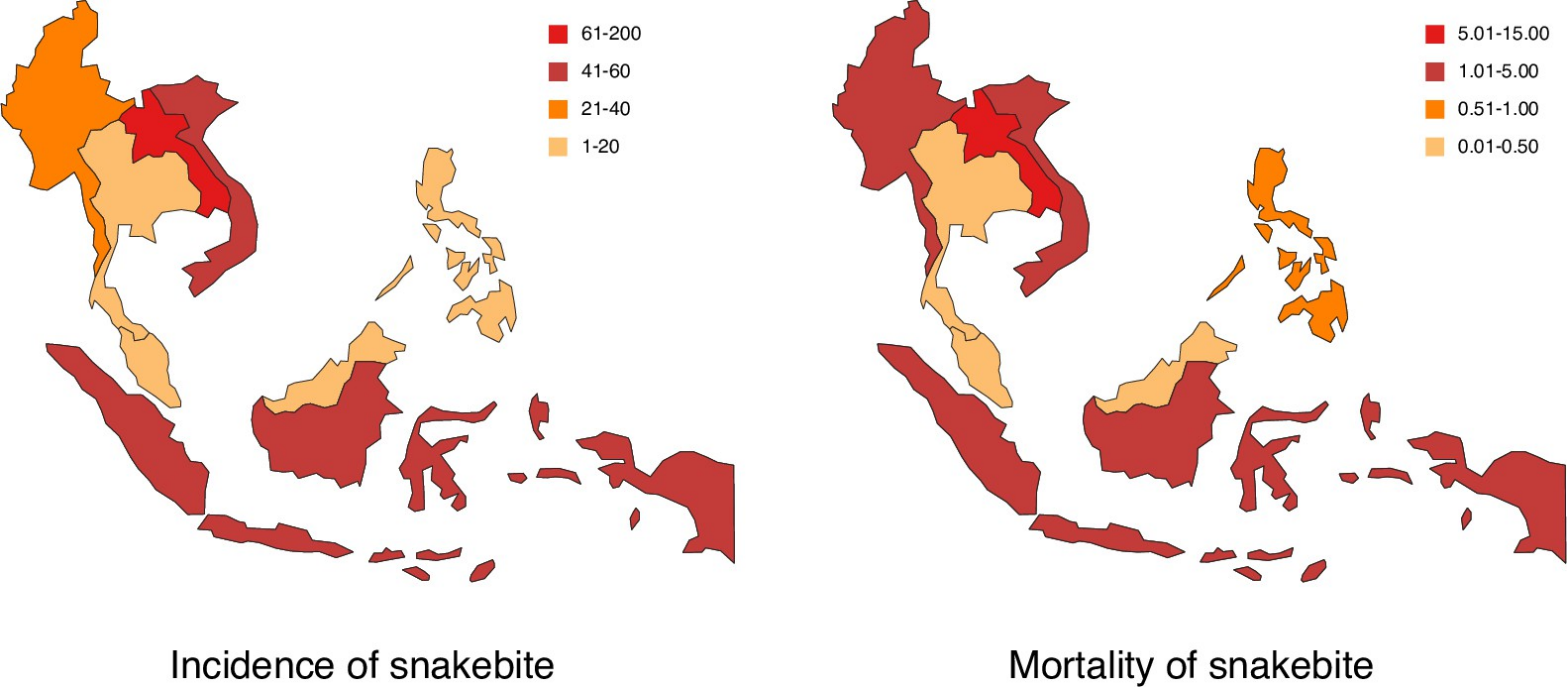
Estimated annual epidemiological burden of snakebite in ASEAN countries. The estimated incidence of snakebite ranged from the lowest in Malaysia (10.68 per 100,000 population) to the highest in Lao PDR (200.00 per 100,000 population). The estimated mortality of snakebite envenoming ranged from the lowest in Thailand (0.006 per 100,000 population) to the highest in Lao PDR (14.04 per 100,000 population). Main sources of information were national statistics and published research for the burden estimation of Malaysia, Thailand, and Myanmar. Published research and anecdotal evidence (local data, and expert opinion) were the main sources of information for the burden estimation of Vietnam, and Lao PDR. Anecdotal evidence was the only source of information for the burden estimation of Indonesia, and Philippines. Made with Natural Earth. Free vector and raster map data @ naturalearthdata.com.

Among 117,575 snakebite victims who were indicated for antivenom treatment (95%CrI 73,790–175,390), there were 954 amputations (95%CrI 383–1,797) and 15,909 deaths (95%CrI 7,592–33,949) following snakebite envenoming. Mortality of snakebite envenoming was estimated at 2.49 per 100,000 population (95%CrI 1.19–5.32), ranging from the lowest in Thailand (0.006 per 100,000 population) to the highest in Lao PDR (14.04 per 100,000 population) (**Fig 2** and **S2 Table**).

**Fig 3:**
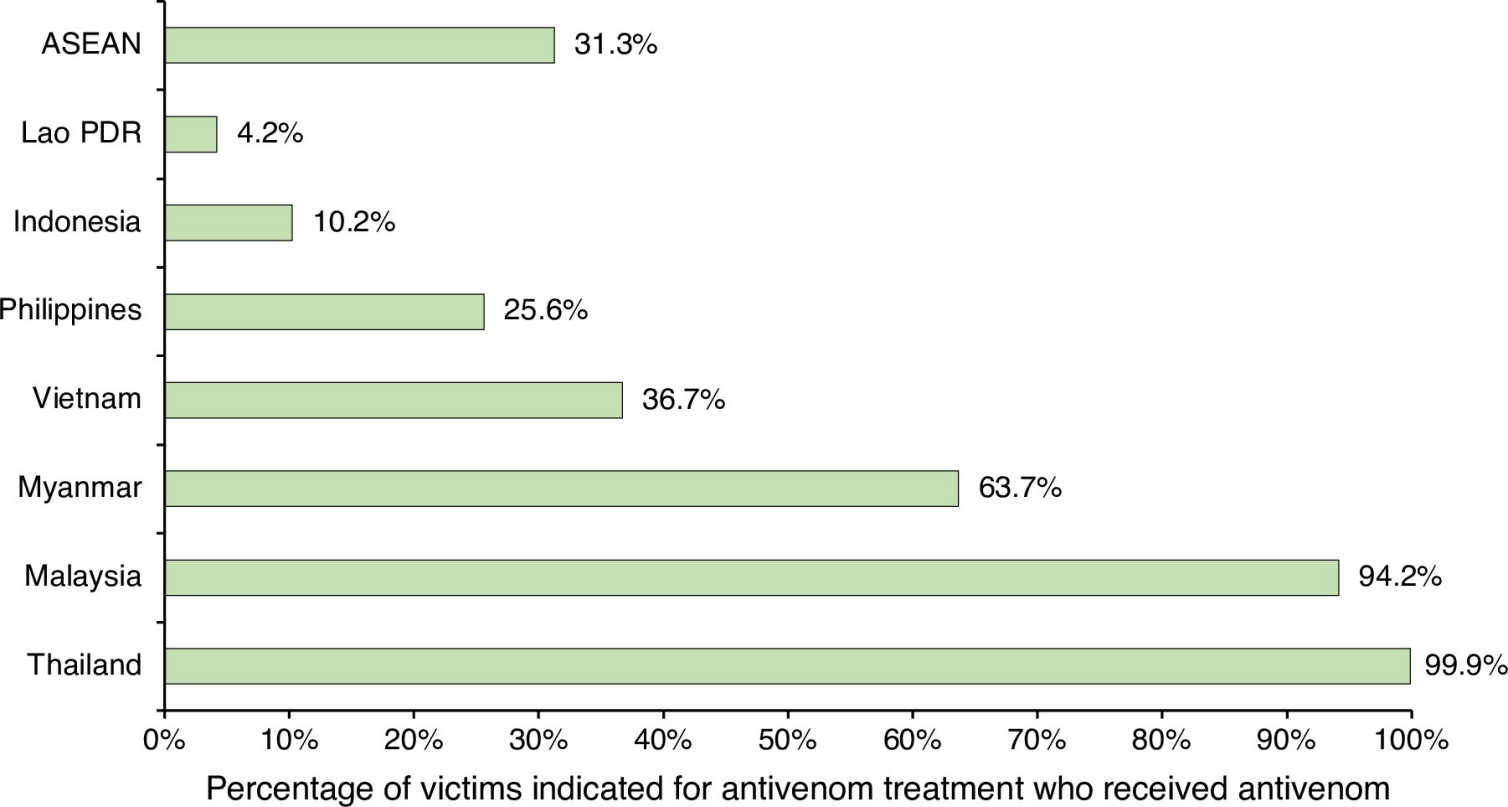
Estimated proportions of snakebite victims treated with antivenom in ASEAN countries. Percentages are estimated from number of snakebite victims treated with antivenom divided by total number of snakebite victims with systemic envenoming who need antivenom; Main sources of information were national statistics and published research for the burden estimation of Malaysia, Thailand, and Myanmar. Published research and anecdotal evidence (local data, and expert opinion) were the main sources of information for the burden estimation of Vietnam, and Lao PDR. Anecdotal evidence was the only source of information for the burden estimation of Indonesia, and Philippines.

It was estimated that 80,813 snakebite victims in ASEAN (31% of victims who were indicated for antivenom treatment) were treated with antivenom, ranging from the lowest in Lao PDR (4.2%) to the highest in Thailand (99.9%) (**Fig 3**).

### Economic burden of snakebite in ASEAN

**Fig 4:**
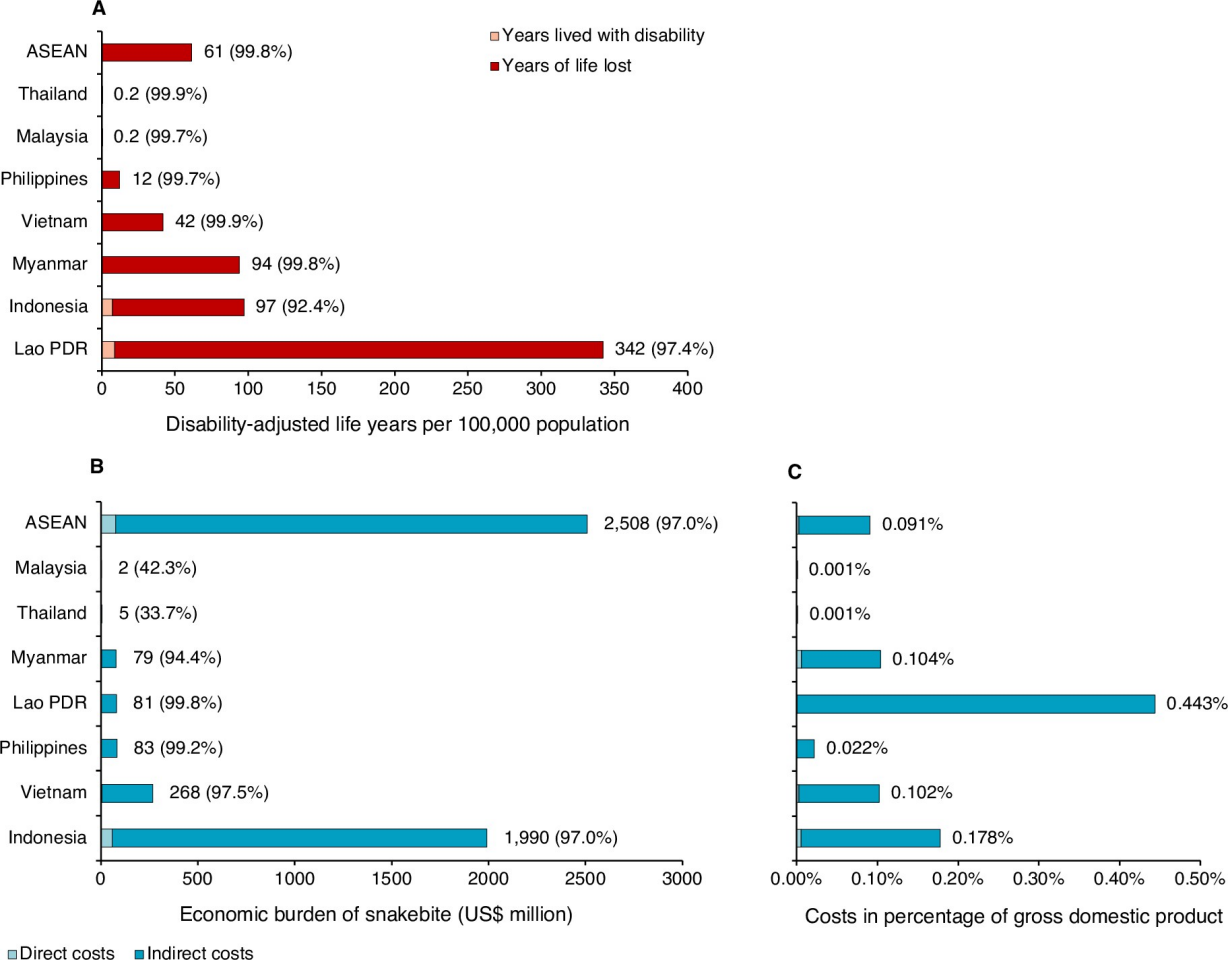
Estimated annual economic and disease burden of snakebite in ASEAN countries. (A) Disease burden of snakebite; data in parentheses are the percentages of disease burden attributable to years of life lost. (B) Costs in million USD; data in parentheses are the percentages of economic burden attributable to indirect costs. (C) Costs in percentage of gross domestic product; Main sources of information were national statistics and published research for the burden estimation of Malaysia, Thailand, and Myanmar. Published research and anecdotal evidence (local data, and expert opinion) were the main sources of information for the burden estimation of Vietnam, and Lao PDR. Anecdotal evidence was the only source of information for the burden estimation of Indonesia, and Philippines. Costs are presented as 2019 USD where 1 USD = 14,147.67 Indonesian Rupees = 51.80 Philippine Pesos = 23,050.24 Vietnamese Dong = 8,679.41 Lao Kip = 1,518.26 Myanmar Kyat. Abbreviation: GDP–gross domestic product; USD—US Dollar.

Annual economic burden of snakebite in ASEAN was estimated at 2.5 billion USD (95%CrI 1.2–5.4 billion USD) which was equivalent to 0.09% (95%CrI 0.04–0.20%) of the GDP (**Table 2** and **Fig 4**). The total costs of snakebite included direct medical costs of 69.0 million USD (95%CrI 49.0–94.8 million USD), direct non-medical costs of 6.5 million USD (95%CrI 4.2–10.3 million USD), and indirect costs of 2.4 billion USD (95%CrI 1.1–5.3 billion USD). The estimated economic burden of snakebite ranged from the lowest in Malaysia (2 million USD) to the highest in Indonesia (1.9 billion USD).

**Table 2:** Estimated annual economic burden (x1,000 USD) of snakebite in ASEAN countries.

	Direct medical costs, x1,000 USD			Direct non-medical costs, x1,000 USD		Indirect costs, x1,000 USD		Total costs, x1,000 USD	Total costs, % of GDP
	Healthcare costs	Antivenom-related costs	Amputation costs	Transportation costs	Additional food costs	Productivity losses during Snakebit episode	Productivity losses due to Premature death		
**Malaysia** ^*^	754 (620–932)	475 (249–758)	0	38 (34–42)	29 (23–40)	366 (289–484)	622 (0–1,866)	2,284 (1,380–3,736)	0.001% (0.000–0.001%)
**Thailand** ^*^	2,027 (1,615–2,531)	1,176 (844–1,506)	0.2 (0–0.6)	58 (54–64)	50 (37–67)	925 (702–1,190)	762 (381–1,333)	4,999 (3,861–6,260)	0.001% (0.001–0.001)
**Indonesia** ^**+**^	51,836 (36,900–70,844)	4,129 (3,727–4,520)	100 (44–178)	1,579 (1,431–1,738)	1,442 (1,027–1,970)	8,752 (6,506–11,566)	1,922,241 (914,489–4,110,887)	1,988,891 (975,513–4,202,049)	0.178% (0.087–0.375%)
**Philippines** ^**+**^	444 (338–578)	147 (130–162)	1 (1–2)	63 (52–76)	46 (35–60)	638 (518–793)	81,905 (40,762–163,735)	83,244 (42,165–165,246)	0.022% (0.011–0.044%)
**Vietnam** ^**¶**^	3,208 (1,090–7,137)	1,094 (447–1,210)	0	853 (299–1,874)	1,463 (494–3,264)	3,801 (1,320–8,251)	257,594 (76,180–690,928)	268,013 (82,106–710,764)	0.102% (0.031–0.271%)
**Lao PDR** ^**¶**^	55 (42–71)	27 (23–32)	12 (2–34)	13 (12–15)	16 (13–20)	427 (361–501)	80,031 (40,573–159,767)	80,583 (41,188–160,291)	0.443% (0.227–0.882%)
**Myanmar** ^*^	1,382 (1,047–1,815)	2,159 (1,910–2,425)	0	474 (417–526)	394 (303–516)	1,208 (952–1,551)	73,569 (44,703–131,172)	79,186 (50,302–136,615)	0.104% (0.066–0.180%)
**Total**	59,706 (41,652–83,950)	9,208 (7,329–10,613)	114 (46–215)	3,078 (2,299–4,335)	3,441 (1,932–5,938)	16,117 (10,648–24,335)	2,416,724 (1,117,087–5,259,687)	2,507,199 (1,196,516–5,384,962)	0.091% (0.043–0.195%)

Estimates are presented as base-case estimates (x 1000 USD) with their 95% credibility interval (in parentheses) based on probabilistic sensitivity analysis. Costs are presented as 2019 USD where 1 USD = 14,147.67 Indonesian Rupees = 51.80 Philippine Pesos = 23,050.24 Vietnamese Dong = 8,679.41 Lao Kip = 1,518.26 Myanmar Kyat. Abbreviation: GDP–gross domestic product; USD—US Dollar

* input parameters were based on national statistics and published literature

^¶^ Input parameters were based on published literature and anecdotal evidence

^+^ Input parameters were based on anecdotal evidence.

The total economic burden of 2.5 billion USD was broken down into hospitalization costs (59.7 million USD; 2.4% of the total economic burden), antivenom-related costs (9.2 million USD; 0.4%), amputation costs (0.1 million USD, 0.005%), transportation costs (3.1 million USD, 0.1%), food costs (3.4 million USD, 0.1%), productivity losses of victims and relatives during snakebite episode (16.1 million USD, 0.6%), and productivity losses due to premature death (2.4 billion USD, 96.4%).

### Disease burden of snakebite in ASEAN

We estimated an annual disease burden of snakebite in ASEAN of 391,979 DALYs (95%CrI 187,261–836,559), which was equivalent to 61 DALYs per 100,000 population (95%CrI 29–131) (**Fig 4 and Tables 1 and S2**). The estimated disease burden of snakebite involved 391,154 YLLs due to death from snakebite envenoming (95%CrI 186,491–835,263; 99.8% of the total DALYs), 330 YLDs for snakebite episode (95%CrI 154–613; 0.08%), and 495 YLDs for amputation (95%CrI 175–1,049; 0.13%). DALYs lost due to snakebite ranged from the lowest in Malaysia (52 DALYs) to the highest in Indonesia (262,888 DALYs).

### Comparison of economic and disease burden per victim with snakebite envenoming across countries

Economic and disease burden per victim with snakebite envenoming was compared across ASEAN countries (**S3 Table**). Mortality rate of snakebite envenoming ranged from the lowest in Thailand (0.001) to the highest in Lao PDR (0.332). Amputation rate of snakebite envenoming ranged from the lowest in Malaysia, Vietnam, and Myanmar (0.000) to the highest in Lao PDR (0.047). DALYs lost due to snakebite envenoming per victim ranged from the lowest in Thailand (0.02 DALYs per victim) to the highest in Lao PDR (8.10 DALYs per victim). Total costs of snakebite envenoming per victim ranged from the lowest in Thailand (861 USD per victim) to the highest in Philippines (47,072 USD per victim).

### Sensitivity analysis

One-way sensitivity analysis found that influential parameters for economic and disease burden were discount rate, probability of death due to snakebite envenoming, relative risk of death when antivenoms are not available, probability of systemic envenoming indicated for antivenom treatment, incidence of snakebite, and length of stay of victims indicated for antivenom treatment (**S1** and **S2 Figs**). When PTSD was incorporated in the model in scenario analysis, the model estimated that there would be 10,293 cases of PTSD (95%CrI 4,651–20,954) with disease burden of 17,458 YLDs (95%CrI 5,869–40,035 YLDs) and productivity losses of 12.7 million USD **(**95%CrI 4.7–27.9 million USD) **(S4 Table**). PTSD following snakebite was found to slightly increased the economic (total costs of 2.52 billion USD; 0.5% increase) and disease burden (405,102 DALYs; 4.5% increase).

## Discussion

To achieve the goal set by the WHO to halve burden of snakebite by 2030, countries should know their current economic and disease burden of snakebite to understand their current standpoint. To our understanding, this is the first study conducted to estimate the economic and disease burden of snakebite in Southeast Asia. The annual economic and disease burden of snakebite in seven ASEAN countries were estimated using a decision analytic model incorporating input parameters from various sources including published literature and local sources to estimate the burden of all snakebite victims regardless of their treatment seeking behavior.

We estimated that annually there were 242,648 snakebite victims (95%CrI 209,810–291,023) of which 15,909 victims (95%CrI 7,592–33,949) were dead and 954 victims (95%CrI 383–1,797) were amputated. The estimated number of snakebite victims and deaths were comparable to the previous estimates in 2007 of approximately 234,000–1,410,000 snakebite victims and 700–18,000 deaths [1]. Annual disease burden of snakebite was estimated at 391,979 DALYs (95%CrI 187,261–836,559). Total costs of snakebite were estimated at 2.5 billion USD (95%CrI 1.2–5.4 billion USD) which were equivalent to 0.09% (95%CrI 0.04–0.20%) of the region’s GDP. The share of the estimated economic burden from snakebite of the country’s GDP ranged from 0.001% in Malaysia to 0.443% in Lao PDR which were remarkably high compared to less than 0.001%. in Iran and Burkina Faso and 0.016% in Sri Lanka [6–9]. The estimated disease burden of snakebite of 391,979 DALYs in seven ASEAN countries (61 DALYs per 100,000 population) was low compared to the previous estimates of 319,874 DALYs in 16 Western African countries (approximately 93 DALYs per 100,000 population) [11] and 1,029,209 DALYs in 41 Sub-Saharan African countries (approximately 120 DALYs per 100,000 population) [10]. This could be partly explained by the differences in the incidence and mortality of snakebite and access to antivenom treatment. Compared to the disease burden of neglected tropical diseases in seven ASEAN countries that were estimated in the Global Burden of Disease 2019 study, snakebite was the second highest burden ranking below dengue (909,899 DALYs) (**S3 Fig**). The disease burdens of malaria (72,844 DALYs) and rabies (66,525 DALYs) were much lower than snakebite [68].

In Malaysia and Thailand, >90% of victims indicated for antivenom could access to it. In contrast, remarkably lower proportions were demonstrated in Lao PDR, Indonesia, Philippines, Vietnam, and Myanmar of which 4–64% antivenom indicated victims were treated with antivenoms. These victims either sought traditional healers or were treated in healthcare facilities but did not receive antivenom due to inadequate supply of antivenom. Consequently, most deaths from snakebite envenoming (99.9%) in ASEAN were from Indonesia, Philippines, Vietnam, Lao PDR, and Myanmar which contributed to high economic and disease burden of premature death from snakebite envenoming. We found that more than 95% of the estimated economic and disease burden was attributed to premature deaths. Treating all snakebite victims who need antivenoms in these countries would save their lives which would result in a tremendous decrease in the burden of snakebite in ASEAN. However, increasing access to antivenom was not only about producing antivenoms but the whole surrounding supporting and management system especially the information system to inform decision making and logistics to efficiently deliver antivenoms even to the farthest healthcare facilities. We previously assessed the situation of snakebite in ASEAN and provided the potential opportunities to improve situation of snakebite in ASEAN to meet the WHO’s target of halving snakebite mortality and morbidity by 2030. These potential opportunities included accurate estimation of antivenom demand, rigorous regulations of antivenom, strengthening the supply chain system, raising public awareness about the importance of treating snakebite envenoming by healthcare professionals, strengthening the health system to ensure appropriate snakebite management and rational use of antivenoms, and expanding collaboration of local and international stakeholders and funders [5].

There were few important limitations of this study worth mentioning. Firstly, Cambodia was not included in this study because we were not able to identify published literature and key informants that could be utilized to estimate the burden of snakebite in Cambodia. It is important to note that Cambodia is one of the countries that imported antivenoms from Thailand, indicating that there were snakebite victims in this country [5]. Secondly, consequences of snakebite included in the model and its sensitivity analysis were limited to death, amputation, and PTSD. Other disabilities such as blindness, malignant ulcers, and pregnancy loss were not included due to a lack of empirical evidence in ASEAN [13]. This warrants future studies in ASEAN to evaluate all relevant consequences and disabilities and associated costs of snakebite to allow better estimation of burden of snakebite. Lastly, there was no nation-wide community and hospital study to comprehensively collect the number of snakebite victims in some of the included countries. Hence, input parameters must be estimated based on non-national studies, local data, and expert opinions, resulting in a wide range of the estimated economic and disease burden of snakebite in ASEAN. This is especially relevant in Lao PDR and Indonesia where snakebite incidences were very high and estimated by local experts. Nevertheless, our findings suggested that there was high burden of snakebite despite the availability of domestically produced antivenoms in the region. We emphasized the importance of funding research to perform a comprehensive data collection on epidemiological and economic burden of snakebite to eventually reveal the true burden of snakebite in ASEAN. These data will yield more accurate information on burden of snakebite to guide decision making in not only the ASEAN but also the WHO to develop global strategies to tackle the problem of snakebite.

## Conclusion

Annual production of 290,000 vials of antivenom in ASEAN were given to only 31% of victims who were indicated for antivenom treatment. Our estimates highlighted the high economic and disease burden of snakebite in ASEAN despite the availability of domestically produced antivenoms. Almost all of the estimated economic and disease burdens were attributed to premature deaths from snakebite envenoming which suggested that the remarkably high burden of snakebite could be averted, especially in countries where large proportions of victims who needed antivenom were not treated with geographically appropriate antivenoms. Strategies should be developed with the goal to improve health outcomes of snakebite victims. However, strategies used to achieve this goal are likely to be complex and different across countries depending on each country’s context and situation such as accurate informatics, rigorous regulations of antivenoms, efficient supply chain, rational use of antivenoms, appropriate treatment seeking behaviors, and good governance to support a strong healthcare system.

## Supporting information

S1 Appendix Justification of input parameters. (DOCX)

S2 Appendix Estimation of economic and disease burden of post-traumatic stress disorder following snakebite envenoming. (DOCX)

S1 Table. Input parameters for estimating economic and disease burden of snakebite in ASEAN countries (DOCX)

S2 Table Estimated annual epidemiological and disease burden of snakebite in 2019 in ASEAN countries. (DOCX)

S3 Table Estimated annual epidemiological and disease burden of snakebite envenoming per case in ASEAN countries. (DOCX)

S4 Table Estimated annual economic and disease burden of post-traumatic stress disorder following snakebite. (DOCX)

S1 Fig One-way sensitivity analysis of economic burden. (DOCX)

S2 Fig One-way sensitivity analysis of disability-adjusted life years (DALYs) of snakebite. (DOCX)

S3 Fig Comparison of annual disease burden of neglected tropical diseases in ASEAN. Estimated disease burden of snakebite from this study (shown in purple) was compared to the disease burden of neglected tropical diseases in seven ASEAN countries that were estimated in the Global Burden of Disease 2019 study.
(DOCX)

## References

[1] Kasturiratne A, Wickremasinghe AR, de Silva N, Gunawardena NK, Pathmeswaran A, Premaratna R, et al. The global burden of snakebite: a literature analysis and modelling based on regional estimates of envenoming and deaths. PLoS Med. 2008;5(11):e218. doi: 10.1371/journal.pmed.0050218 18986210 PMC2577696

[2] World Health Organization. Snakebite envenoming: Geneva: World Health Organization; 2018 [February 26, 2019]. Available from: https://www.who.int/news-room/fact-sheets/detail/snakebite-envenoming.

[3] World Health Organization. Snakebite envenoming: a strategy for prevention and control [Internet]. 2019. Available from: https://apps.who.int/iris/bitstream/handle/10665/324838/9789241515641-eng.pdf. 10.1016/S2214-109X(19)30225-6 31129124

[4] Association of Southeast Asian Nations. ASEAN Member States [Internet]. Available from: https://asean.org/asean/asean-member-states/.

[5] Patikorn C, Ismail AK, Abidin SAZ, Blanco FB, Blessmann J, Choumlivong K, et al. Situation of snakebite, antivenom market, and access to antivenom in ASEAN countries. BMJ Global Health. 2022;0:e007639. doi: 10.1136/bmjgh-2021-007639 35296460 PMC8928241

[6] Patikorn C, Leelavanich D, Ismail AK, Othman I, Taychakhoonavudh S, Chaiyakunapruk N. Global systematic review of cost of illness and economic evaluation studies associated with snakebite. J Glob Health. 2020;10(2). doi: 10.7189/jogh.10.020415 33312499 PMC7719278

[7] Gampini S, Nassouri S, Chippaux J-P, Semde R. Retrospective study on the incidence of envenomation and accessibility to antivenom in Burkina Faso. Journal of Venomous Animals and Toxins including Tropical Diseases. 2016;22(1):10. doi: 10.1186/s40409-016-0066-7 26985188 PMC4793557

[8] Mashhadi I, Kavousi Z, Peymani P, Salman Zadeh Ramhormozi S, Keshavarz K. Economic burden of scorpion sting and snake bite from a social perspective in Iran. Shiraz E Medical Journal. 2017;18(8). doi: 10.5812/semj.57573

[9] Kasturiratne A, Pathmeswaran A, Wickremasinghe AR, Jayamanne SF, Dawson A, Isbister GK, et al. The socio-economic burden of snakebite in Sri Lanka. PLoS Negl Trop Dis. 2017;11(7):e0005647. Epub 2017/07/07. doi: 10.1371/journal.pntd.0005647 ; PubMed Central PMCID: PMC5500261. 28683119 PMC5500261

[10] Halilu S, Iliyasu G, Hamza M, Chippaux JP, Kuznik A, Habib AG. Snakebite burden in Sub-Saharan Africa: estimates from 41 countries. Toxicon. 2019;159:1–4. Epub 2018/12/31. doi: 10.1016/j.toxicon.2018.12.002 . 30594637

[11] Habib AG, Kuznik A, Hamza M, Abdullahi MI, Chedi BA, Chippaux JP, et al. Snakebite is Under Appreciated: Appraisal of Burden from West Africa. PLoS Negl Trop Dis. 2015;9(9):e0004088. Epub 2015/09/24. doi: 10.1371/journal.pntd.0004088 ; PubMed Central PMCID: PMC4580425. 26398046 PMC4580425

[12] Ahmed S, Koudou GB, Bagot M, Drabo F, Bougma WR, Pulford C, et al. Health and economic burden estimates of snakebite management upon health facilities in three regions of southern Burkina Faso. PLoS neglected tropical diseases. 2021;15(6):e0009464. doi: 10.1371/journal.pntd.0009464 34153048 PMC8248599

[13] Habib AG, Lamorde M, Dalhat MM, Habib ZG, Kuznik A. Cost-effectiveness of antivenoms for snakebite envenoming in Nigeria. PLoS Negl Trop Dis. 2015;9(1):e3381. Epub 2015/01/09. doi: 10.1371/journal.pntd.0003381 ; PubMed Central PMCID: PMC4287484. 25569252 PMC4287484

[14] Hamza M, Idris MA, Maiyaki MB, Lamorde M, Chippaux JP, Warrell DA, et al. Cost-Effectiveness of Antivenoms for Snakebite Envenoming in 16 Countries in West Africa. PLoS Negl Trop Dis. 2016;10(3):e0004568. Epub 2016/03/31. doi: 10.1371/journal.pntd.0004568 ; PubMed Central PMCID: PMC4814077. 27027633 PMC4814077

[15] Shafie NA, Fauzi H, Wahab M, Senek M, Ismail A. The prevalence of hypersensitivity reactions to snake antivenoms administered in sultanah nur zahirah hospital from 2013 to 2016. Med J Malaysia. 2020;75(3):217. 32467535

[16] Thiansookon A, Rojnuckarin P. Low incidence of early reactions to horse-derived F (ab′) 2 antivenom for snakebites in Thailand. Acta tropica. 2008;105(2):203–5. doi: 10.1016/j.actatropica.2007.09.007 17996842

[17] Adiwinata R, Nelwan EJ. Snakebite in Indonesia. Acta Med Indones. 2015;47(4):358–65. Epub 2016/03/05. . 26932707

[18] Watt G, Padre L, Tuazon ML, Hayes CG. Bites by the Philippine cobra (Naja naja philippinensis): an important cause of death among rice farmers. Am J Trop Med Hyg. 1987;37(3):636–9. doi: 10.4269/ajtmh.1987.37.636 3688317

[19] Vongphoumy I, Chanthilat P, Vilayvong P, Blessmann J. Prospective, consecutive case series of 158 snakebite patients treated at Savannakhet provincial hospital, Lao People’s Democratic Republic with high incidence of anaphylactic shock to horse derived F (ab’) 2 antivenom. Toxicon. 2016;117:13–21. doi: 10.1016/j.toxicon.2016.03.011 26995210

[20] Mahmood MA, Halliday D, Cumming R, Thwin K-T, Zu Kyaw MM, White J, et al. Snakebite incidence in two townships in Mandalay Division, Myanmar. PLoS Negl Trop Dis. 2018;12(7):e0006643. doi: 10.1371/journal.pntd.0006643 29985919 PMC6053239

[21] Thang VV, Bao TQQ, Tuyen HD, Krumkamp R, Hai LH, Dang NH, et al. Incidence of snakebites in Can Tho Municipality, Mekong Delta, South Vietnam—Evaluation of the responsible snake species and treatment of snakebite envenoming. PLoS Negl Trop Dis. 2020;14(6):e0008430. doi: 10.1371/journal.pntd.0008430 32555599 PMC7323996

[22] Sivaganabalan R, Ismail AK, Salleh MS, Mohan K, Tan CH, Adnan A. Guideline on the Management of Snakebites. Ministry of Health Malaysia; 2017.

[23] National Health Security Office, Thailand. Report on the creation of the National Health Security for fiscal year BE 2562 (AD 2019). Bangkok, Thailand; 2019.

[24] Longbottom J, Shearer FM, Devine M, Alcoba G, Chappuis F, Weiss DJ, et al. Vulnerability to snakebite envenoming: A global mapping of hotspots. The Lancet. 2018;392(10148):673–84. doi: 10.1016/S0140-6736(18)31224-8 30017551 PMC6115328

[25] Mitrakul C, Dhamkrong-At A, Futrakul P, Thisyakorn C, Vongsrisart K, Varavithya C, et al. Clinical features of neurotoxic snake bite and response to antivenom in 47 children. Am J Trop Med Hyg. 1984;33(6):1258–66. Epub 1984/11/01. doi: 10.4269/ajtmh.1984.33.1258 . 6507733

[26] Wongtongkam N, Wilde H, Sitthi-Amorn C, Ratanabanangkoon K. A study of Thai cobra (Naja kaouthia) bites in Thailand. Military medicine. 2005;170(4):336–41. doi: 10.7205/milmed.170.4.336 15916306

[27] Wongtongkam N, Wilde H, Sitthi-Amorn C, Ratanabanangkoon K. A study of 225 Malayan pit viper bites in Thailand. Military Medicine. 2005;170(4):342–8. doi: 10.7205/milmed.170.4.342 15916307

[28] Hutton RA, Looareesuwanzj S, Ho M, Silamut K, Chanthavanich P, Karbwang J, et al. Arboreal green pit vipers (genus Trimeresurus) of south-east Asia: Bites by T. albolabris and T. macrops in Thailand and a review of the literature. Trans R Soc Trop Med Hyg. 1990;84(6):866–74. doi: 10.1016/0035-9203(90)90111-q 2096527

[29] Mitrakul C, Juzi U, Pongrujikorn W. Antivenom therapy in Russell’s viper bite. Am J Clin Pathol. 1991;95(3):412–7. Epub 1991/03/01. doi: 10.1093/ajcp/95.3.412 . 1996552

[30] Viravan C, Looareesuwan S, Kosakam W, Wuthiekanun V, McCarthy CJ, Stimson AF, et al. A national hospital-based survey of snakes responsible for bites in Thailand. Trans R Soc Trop Med Hyg. 1992;86(1):100–6. doi: 10.1016/0035-9203(92)90463-m 1566285

[31] Rojnuckarin P, Mahasandana S, Intragumthornchai T, Sutcharitchan P, Swasdikul D. Prognostic factors of green pit viper bites. Am J Trop Med Hyg. 1998;58(1):22–5. Epub 1998/02/06. doi: 10.4269/ajtmh.1998.58.22 . 9452286

[32] Rojnuckarin P, Intragumtornchai T, Sattapiboon R, Muanpasitporn C, Pakmanee N, Khow O, et al. The effects of green pit viper (Trimeresurus albolabris and Trimeresurus macrops) venom on the fibrinolytic system in human. Toxicon. 1999;37(5):743–55. Epub 1999/04/29. doi: 10.1016/s0041-0101(98)00214-1 . 10219986

[33] Chotenimitkhun R, Rojnuckarin P. Systemic antivenom and skin necrosis after green pit viper bites. Clin Toxicol (Phila). 2008;46(2):122–5. Epub 2008/02/09. doi: 10.1080/15563650701266826 . 18259959

[34] Laohawiriyakamol S, Sangkhathat S, Chiengkriwate P, Patrapinyokul S. Surgery in management of snake envenomation in children. World J Pediatr. 2011;7(4):361–4. Epub 2011/08/31. doi: 10.1007/s12519-011-0282-8 . 21877258

[35] Tongpoo A, Sriapha C, Pradoo A, Udomsubpayakul U, Srisuma S, Wananukul W, et al. Krait envenomation in Thailand. Therapeutics and Clinical Risk Management. 2018;14:1711–7. doi: 10.2147/TCRM.S169581 30271155 PMC6145358

[36] Thumtecho S, Tangtrongchitr T, Srisuma S, Kaewrueang T, Rittilert P, Pradoo A, et al. Hematotoxic manifestations and management of green pit viper bites in Thailand. Therapeutics and Clinical Risk Management. 2020;16:695. doi: 10.2147/TCRM.S261303 32801726 PMC7398752

[37] Malasit P, Warrell DA, Chanthavanich P, Viravan C, Mongkolsapaya J, Singhthong B, et al. Prediction, prevention, and mechanism of early (anaphylactic) antivenom reactions in victims of snake bites. British Medical Journal (Clinical research ed). 1986;292(6512):17–20. doi: 10.1136/bmj.292.6512.17 3080048 PMC1338972

[38] Pongpit J, Limpawittayakul P, Juntiang J, Akkawat B, Rojnuckarin P. The role of prothrombin time (PT) in evaluating green pit viper (Cryptelytrops sp) bitten patients. Trans R Soc Trop Med Hyg. 2012;106(7):415–8. doi: 10.1016/j.trstmh.2012.04.003 22627102

[39] Le Khac Q. Clinical evaluation of snakebites in Vietnam: A study from Cho Ray hospital. 2004.

[40] Habib AG, Warrell DA. Antivenom therapy of carpet viper (Echis ocellatus) envenoming: effectiveness and strategies for delivery in West Africa. Toxicon. 2013;69:82–9. doi: 10.1016/j.toxicon.2013.01.002 23339853

[41] Jiranantakan T, Pantumongkol W, Uansri S, Wisaiprom J, Tantivess S, Leelahavarong P, et al. Budget Impact, Output and Outcome Analysis of Thailand National Antidote Project [Internet]. Health Intervention and Technology Assessment Program, Thailand; 2019 [cited 2020 August 27]. Available from: https://www.hitap.net/en/research/174746.

[42] Pochanugool C, Limthongkul S, Wilde H. Management of Thai cobra bites with a single bolus of antivenin. Wilderness and Environmental Medicine. 1997;8(1):20–3. doi: 10.1580/1080-6032(1997)008[0020:motcbw]2.3.co;2 11990132

[43] Trishnananda M, Oonsombat P, Dumavibhat B, Yongchaiyudha S, Boonyapisit V. Clinical manifestations of cobra bite in the Thai farmer. Am J Trop Med Hyg. 1979;28(1):165–6. doi: 10.4269/ajtmh.1979.28.165 434309

[44] Buranasin P. Snakebites at Maharat Nakhon Ratchasima Regional Hospital. The Southeast Asian journal of tropical medicine and public health. 1993;24(1):186–92. 8362295

[45] Pochanugool C, Wilde H, Bhanganada K, Chanhome L, Cox MJ, Chaiyabutr N, et al. Venomous snakebite in Thailand II: Clinical experience. Military Medicine. 1998;163(5):318–23. doi: 10.1093/milmed/163.5.318 9597849

[46] Salomon JA, Haagsma JA, Davis A, de Noordhout CM, Polinder S, Havelaar AH, et al. Disability weights for the Global Burden of Disease 2013 study. The Lancet Global Health. 2015;3(11):e712–e23. doi: 10.1016/S2214-109X(15)00069-8 26475018

[47] Attorney General’s Chambers Malaysia. Fees (Medical) (Cost of Services) Order 2014. Federal Government Gazette; 2014.

[48] Menteri Kesehatan Republik Indonesia. Peraturan Menteri Kesehatan Republik Indonesia Nomor 63 Tahun 2014 tentang Pengadaan Obat Berdasarkan Katalog Elektronik (E-catalogue). 2014.

[49] Edillo FE, Halasa YA, Largo FM, Erasmo JNV, Amoin NB, Alera MTP, et al. Economic cost and burden of dengue in the Philippines. Am J Trop Med Hyg. 2015;92(2):360–6. doi: 10.4269/ajtmh.14-0139 25510723 PMC4347342

[50] Department of Health, Republic of the Philippines. Drug Price Reference Index [Internet]. [cited 2021 May 10]. Available from: https://dpri.doh.gov.ph/index.php?page=search.

[51] Maternal and Reproductive Health Division, Department of Public Health Myanmar. Costed implementation plan to meet family planning 2020 commitments of Myanmar. Strategic prioritization of implementation 2018–2020 [Internet]. 2018. Available from: https://www.familyplanning2020.org/sites/default/files/myanmar_cip_2018.10.pdf.

[52] Flessa S, Dung NT. Costing of services of Vietnamese hospitals: identifying costs in one central, two provincial and two district hospitals using a standard methodology. The international journal of health planning and management. 2004;19(1):63–77. doi: 10.1002/hpm.747 15061290

[53] Riewpaiboon A. Standard cost lists for health economic evaluation in Thailand. Journal of the Medical Association of Thailand = Chotmaihet Thangphaet. 2014;97:S127–34. 24964710

[54] Pharmaceutical Services Programme, Ministry of Health Malaysia,. Consumer Price Guide [Internet]. Available from: https://www.pharmacy.gov.my/v2/en/apps/drug-price.

[55] Chong HY, Lim YH, Prawjaeng J, Tassaneeyakul W, Mohamed Z, Chaiyakunapruk N. Cost-effectiveness analysis of HLA-B* 58: 01 genetic testing before initiation of allopurinol therapy to prevent allopurinol-induced Stevens–Johnson syndrome/toxic epidermal necrolysis in a Malaysian population. Pharmacogenetics and genomics. 2018;28(2):56–67. doi: 10.1097/FPC.0000000000000319 29176400

[56] Riewpaiboon A. Economic burden of hand, foot, and mouth disease in Vietnam; An evidence for priority setting and efficiency management.

[57] Vodicka E, Zimmermann M, Lopez AL, Silva MW, Gorgolon L, Kohei T, et al. Japanese encephalitis vaccination in the Philippines: A cost-effectiveness analysis comparing alternative delivery strategies. Vaccine. 2020;38(13):2833–40. doi: 10.1016/j.vaccine.2020.02.018 32085954 PMC7068699

[58] Division of Pharmaceutical Sciences, Ministry of Health Malaysia (MOH). Pharmacoeconomic guideline for Malaysia. Putrajaya, Malaysia: 2012.

[59] Teerawattananon Y. Guidelines for health technology assessment in Thailand (second edition). J Med Assoc Thai. 2014;97(5):S4–9. 24964693

[60] Indonesian Health Technology Assessment Committee MoHotRoI. Health Technology Assessment (HTA) guideline.

[61] World Bank. GDP per capita (current LCU) [Internet]. 2019. Available from: https://data.worldbank.org/indicator/NY.GDP.PCAP.CN.

[62] World Bank. GDP per capita growth (annual %) [Internet]. 2019. Available from: https://data.worldbank.org/indicator/NY.GDP.PCAP.KD.ZG.

[63] Mathers CD, Vos T, Lopez AD, Salomon J, Ezzati M. National burden of disease studies: a practical guide. Geneva: World Health Organization. 2001.

[64] Khosrojerdi H, Amini M. Acute and delayed stress symptoms following snakebite. Asia Pacific Journal of Medical Toxicology. 2013;2(4):140–4.

[65] Waiddyanatha S, Silva A, Siribaddana S, Isbister GK. Long-term effects of snake envenoming. Toxins. 2019;11(4):193. doi: 10.3390/toxins11040193 30935096 PMC6521273

[66] Kessler RC, Aguilar-Gaxiola S, Alonso J, Benjet C, Bromet EJ, Cardoso G, et al. Trauma and PTSD in the WHO world mental health surveys. European journal of psychotraumatology. 2017;8(sup5):1353383. doi: 10.1080/20008198.2017.1353383 29075426 PMC5632781

[67] Ferry FR, Brady SE, Bunting BP, Murphy SD, Bolton D, O’Neill SM. The economic burden of PTSD in Northern Ireland. Journal of traumatic stress. 2015;28(3):191–7. doi: 10.1002/jts.22008 25990825

[68] Vos T, Lim SS, Abbafati C, Abbas KM, Abbasi M, Abbasifard M, et al. Global burden of 369 diseases and injuries in 204 countries and territories, 1990–2019: a systematic analysis for the Global Burden of Disease Study 2019. The Lancet. 2020;396(10258):1204–22. doi: 10.1016/S0140-6736(20)30925-9 33069326 PMC7567026

