# Supplementary material for: Estimating economic and disease burden of snakebite in ASEAN countries using a decision analytic model
Source: PLoS Negl Trop Dis. 2022 Sep 28;16(9):e0010775. doi: 10.1371/journal.pntd.0010775 (PMC9518918; doi:10.1371/journal.pntd.0010775)
Supplement: S1 Appendix — (DOCX) [file pntd.0010775.s001.docx]

**SUPPLEMENTARY MATERIAL**

Estimating economic and disease burden of snakebite in ASEAN countries using a decision analytic model

**S1 Appendix. Justification of input parameters.**

Country-specific input parameters were sought from various sources, including published literature, data from the country’s Ministry of Health, local data, and expert opinion. When parameters were available from multiple sources, the apply value were selected based on the most recent evidence and the representativeness of the data that covered the highest number of subjects in the following order of priority; (1) Published community-based national data, (2) Published hospital-based national data, (3) Local national data (community/hospital based, (4) Published community-based subnational data, (5) Local community based subnational data, (6) Published subnational hospital data, and (7) Expert opinion.[1] When data of the country were not available, the parameters were borrowed from other countries.

An in-depth interview with key informants who were experts in snakebite in ASEAN countries was also conducted to confirm the retrieved parameters, refer to potential sources of information that might not be publicly available, and ask for their opinion when data were not available. The input parameters were validated through triangulation of data from literature and interview. Justification of input parameters for each country was described below.

**Malaysia**

**Incidence of snakebite**
- Number of snakebite patients treated in healthcare facilities in 2014[2] = 3,006 cases
- Number of population in 2014[3] = 29,866,559 people

- Number of vulnerable people living within the range of one or more medically important venomous snake species, for which no effective therapy exists, and with a travel time of more than 3 hours from urban centers in 2017[4] = 1,790,903 people

- Number of population in 2017[3] = 31,105,028 people

- Proportion of people who could not access to healthcare facilities and would seek traditional healers in 2017 = $\frac{1,790,903}{31,105,028}$ = 0.0576

- Number of people who could not access to healthcare facilities and would seek traditional healers in 2014 = 0.0576 x 29,866,559 = 1,719,597 people

- Number of people who could access to healthcare facilities = 31,105,028 – 1,719,597 = 28,146,962 people
- Incidence of snakebite patients treated in healthcare facilities in 2014 = $\frac{3,006}{28,146,962}$ = 10.68 cases per 100,000 population per year

- Number of snakebite victims who could not access to healthcare facilities and sought traditional healers in 2014 = 0.0001068 x 1,719,597 = 184 cases

- Total number of snakebite victims in 2014 = 3,006 + 184 = 3,190 cases

**Treatment seeking behavior**

- Total number of snakebite victims in 2014 = 3,190 cases

- Number of snakebite patients treated in healthcare facilities in 2014[2] = 3,006 cases

- Number of snakebite victims who could not access to healthcare facilities and sought traditional healer in 2014 = 184 cases

- Proportion of snakebite victims in healthcare facilities who sought traditional healer first then switched to conventional treatment (local data) = $\frac{1}{268}$ = 0.0037 (95%CI 0.0001 to 0.0206)

- Number of snakebite victims who sought traditional healer first then switched to conventional treatment in 2014 = 0.0037 x 3,006 = 11 cases

- Number of snakebite victims who sought conventional treatment only = 3,006 – 11 = 2,995

- Proportion of snakebite victims who sought conventional treatment only = $\frac{2,995}{3,190}$ = 0.939

- Proportion of snakebite victims who sought traditional healer first = 1 – 0.939 = 0.061

- Proportion of snakebite victims who sought traditional healer first then switched to conventional treatment = $\frac{11}{195}$ = 0.058

- Proportion of snakebite victims who sought only traditional healer = 1 – 0.058 = 0.942

**Mortality of snakebite**

- Probability of systemic envenoming needed antivenom treatment in 2017[5] = $\frac{13}{92}$ = 0.1413 (95%CI; 0.077 to 0.230)
- Number of patients with systemic envenomings treated in healthcare facilities in 2014 = 3,006 x 0.1413 = 421 cases

- Number of deaths from systemic envenoming treated in healthcare facilities in 2014[2] = 1 death
- Probability of death of systemic envenoming treated in healthcare facilities in 2014 = $\frac{1}{421}$ = 0.002 (95%CI 0.001 to 0.013)

- Relative risk of death in snakebite envenoming without antivenom treatment compared to with antivenom treatment[6]= 2.33

- Probability of death of systemic envenoming treated without antivenom treatment in healthcare facilities in 2014 = 0.002 x 2.33 = 0.005

- Probability of death of systemic envenoming not treated in healthcare facilities in 2014 = 0.013 x 2.33 = 0.030

**Hospitalization costs for snakebite victims with systemic envenoming**

- Hospitalization costs comprise of inpatient department services, laboratory, tetanus toxoid, and wound dressing.

| **Item** | **Quantity** | **Price (MYR)** | **Cost (MYR)** |
| --- | --- | --- | --- |
| **Hospitalization costs, total** |  |  | **3,111.03** |
| **Inpatient department services** | **6.1[5]** | **109.96[7]** | **666.55** |
| **Laboratory for systemic envenoming, average** |  |  | **1,907.74** |
| Proportion of hematotoxic to neurotoxic snakes | 6:4^*^ |  |  |
| Laboratory for systemic envenoming, hematotoxic snakes |  |  | 2,622.46 |
| - Coagulation profile | 12^*^ | 126.45[7] | 1,517.40 |
| - Complete blood count | 12^*^ | 43.98[7] | 527.79 |
| - Urine analysis | 2^*^ | 32.99[7] | 65.97 |
| - Electrolyte | 3^*^ | 11.00[7] | 32.99 |
| - Blood urea nitrogen | 4^*^ | 11.00[7] | 43.98 |
| - Creatinine | 1^*^ | 38.48[7] | 38.48 |
| - Electrocardiogram | 1^*^ | 87.97[7] | 87.97 |
| - Creatine kinase | 12^*^ | 21.99[7] | 263.90 |
| - Bacterial culture | 1^*^ | 43.98[7] | 43.98 |
| Laboratory for systemic envenoming, neurotoxic snakes |  |  | 835.67 |
| - Coagulation profile | 1^*^ | 126.45[7] | 126.45 |
| - Complete blood count | 3^*^ | 43.98[7] | 131.95 |
| - Urine analysis | 2^*^ | 32.99[7] | 65.97 |
| - Electrolyte | 3^*^ | 11.00[7] | 32.99 |
| - Blood urea nitrogen | 4^*^ | 11.00[7] | 43.98 |
| - Creatinine | 1^*^ | 38.48[7] | 38.48 |
| - Electrocardiogram | 1^*^ | 87.97[7] | 87.97 |
| - Creatine kinase | 12^*^ | 21.99[7] | 263.90 |
| - Bacterial culture | 1^*^ | 43.98[7] | 43.98 |
| **Tetanus toxoid** |  |  | **3.50** |
| - Tetanus toxoid | 1^*^ | 3.15[8] | 3.15 |
| - Needle | 1^*^ | 0.04[9] | 0.04 |
| - Syringe | 1^*^ | 0.31[9] | 0.31 |
| **Wound dressing** | **6.1[5]** | **87.97[10]** | **533.24** |

Note: * – expert opinion; MYR – Malaysian Ringgit where 4.14 MYR = 1 United States Dollar.

**Hospitalization costs for victims without snakebite envenoming**

- Hospitalization costs comprise of inpatient department services, laboratory, tetanus toxoid, and wound dressing.

| **Item** | **Quantity** | **Price (MYR)** | **Cost (MYR)** |
| --- | --- | --- | --- |
| **Hospitalization costs, total** |  |  | **619.25** |
| **Inpatient department services** | 1^*^ | **109.96[7]** | **109.96** |
| **Laboratory for snakebite without systemic envenoming** |  |  | **417.83** |
| - Coagulation profile | 1^*^ | 126.45[7] | 126.45 |
| - Complete blood count | 1^*^ | 43.98[7] | 43.98 |
| - Urine analysis | ^1*^ | 32.99[7] | 32.99 |
| - Electrolyte | ^1*^ | 11.00[7] | 11.00 |
| - Blood urea nitrogen | ^1*^ | 11.00[7] | 11.00 |
| - Creatinine | 1^*^ | 38.48[7] | 38.48 |
| - Electrocardiogram | 1^*^ | 87.97[7] | 87.97 |
| - Creatine kinase | 11^*^ | 21.99[7] | 21.99 |
| - Bacterial culture | 1^*^ | 43.98[7] | 43.98 |
| **Tetanus toxoid** |  |  | **3.50** |
| - Tetanus toxoid | 1^*^ | 3.15[8] | 3.15 |
| - Needle | 1^*^ | 0.04[9] | 0.04 |
| - Syringe | 1^*^ | 0.31[9] | 0.31 |
| **Wound dressing** | 1^*^ | **87.97[10]** | **87.97** |

Note: * – expert opinion; MYR – Malaysian Ringgit where 4.14 MYR = 1 United States Dollar.

**Antivenom treatment costs**

- Antivenom treatment costs comprise of antivenom, and antivenom administration.

| **Item** | **Quantity** | **Price (MYR)** | **Cost (MYR)** |
| --- | --- | --- | --- |
| **Antivenom treatment costs, total** |  |  | **4,131.41** |
| **Antivenom, average** |  |  | **4,109.06** |
| Proportion of hematotoxic to neurotoxic snakes | 6:4^*^ |  |  |
| Antivenom, hematotoxic snakes | 4^*^ | 790.20[11] | 3,160.81 |
| Antivenom, neurotoxic snakes | 7^*^ | 790.20[11] | 5,531.43 |
| **Antivenom administration** |  |  | **22.35** |
| - Needle | 1^*^ | 0.04[9] | 0.04 |
| - Syringe | 1^*^ | 0.31[9] | 0.31 |
| - 0.9% NaCl 100 mL | 1^*^ | 2.31[11] | 2.31 |
| - IV set | 1^*^ | 19.69[9] | 19.69 |

Note: * – expert opinion; MYR – Malaysian Ringgit where 4.14 MYR = 1 United States Dollar.

**Adverse reaction management costs**

| **Item** | **Quantity** | **Price (MYR)** | **Cost (MYR)** |
| --- | --- | --- | --- |
| **Adverse reaction management costs, total** |  |  | **16.29** |
| - Chlorpheniramine | 1^*^ | 2.71[11] | 2.71 |
| - Adrenaline | 1^*^ | 3.64[11] | 3.64 |
| - Hydrocortisone | 1^*^ | 8.89[11] | 8.89 |
| - Needle | 3^*^ | 0.04[9] | 0.13 |
| - Syringe | 3^*^ | 0.31[9] | 0.92 |

Note: * – expert opinion; MYR – Malaysian Ringgit where 4.14 MYR = 1 United States Dollar.

**Thailand**

**Incidence of snakebite**
- Number of snakebite patients treated with antivenom in 2019[12] = 5,160 cases
- Number of population in 2019[3] = 64,929,153 people

- Number of vulnerable people living within the range of one or more medically important venomous snake species, for which no effective therapy exists, and with a travel time of more than 3 hours from urban centers in 2017[4] = 77,295 people

- Number of population in 2017[3] = 69,209,858 people

- Proportion of people who could not access to healthcare facilities and would seek traditional healers in 2017 = $\frac{77,295}{69,209,858}$ = 0.00119 (95%CI 0.00118 to 0.00120)

- Number of people who could not access to healthcare facilities and would seek traditional healers in 2019 = 0.00119 x 69,209,858 = 82,886 people

- Number of people who could access to healthcare facilities = 69,209,858 – 82,886 = 69,542,696 people

- Probability of systemic envenoming required antivenom derived from meta-analysis[13-26] = 0.59 (95%CI 0.42 to 0.74)

Study | ES [95% Conf. Interval]

---------------------+---------------------------------------------------

Mitrakul (1984) | 0.9362 0.8246 0.9866

Malasit (1986) | 0.8250 0.7238 0.9009

Hutton (1990) | 0.7083 0.4891 0.8738

Mitrakul (1991) | 0.6250 0.2449 0.9148

Viravan (1992) | 0.1923 0.1682 0.2183

Buranasin (1993) | 0.3832 0.2908 0.4822

Rojnuckarin (1996) | 0.7122 0.6551 0.7647

Rojnuckarin (1998) | 0.7085 0.6505 0.7619

Rojnuckarin (1999) | 0.1304 0.0494 0.2626

Wongtongkam (2005) | 0.8880 0.8192 0.9374

Thiansookon (2008) | 0.6649 0.6151 0.7121

Chotenimitkhun (2008) | 0.3292 0.2705 0.3922

Laohawiriyakamol (2011)| 0.3793 0.2551 0.5163

Pingpit (2012) | 0.1237 0.0656 0.2061

Tongpoo (2018) | 0.8205 0.7172 0.8983

Thumtecho (2020) | 0.6389 0.5805 0.6944

---------------------+---------------------------------------------------

Random pooled ES | 0.5928 0.4231 0.7429

---------------------+---------------------------------------------------

LR test: RE vs FE Model chi^2 = 765.8970 (d.f. = 14) p = 0.0000

Estimate of between-study variance Tau^2 = 1.5260

Test of ES=0 : z= 1.0732 p = 0.2832

- Number of snakebite patients treated in healthcare facilities = = $\frac{5,160}{0.5928}$ = 8,704 cases
- Incidence of snakebite patients treated in healthcare facilities in 2019 = $\frac{8,704}{64,929,153}$ = 12.52 cases per 100,000 population per year

- Number of snakebite victims who could not access to healthcare facilities and sought traditional healers in 2019 = 0.0001252 x 82,886 = 11 cases

- Total number of snakebite victims in 2019 = 8,704 + 11 = 8,715 cases

**Treatment seeking behavior**

- Proportion of snakebite victims in healthcare facilities who sought traditional healer first then switched to conventional treatment derived from meta-analysis[13, 19, 27] = 0.0339 (95%CI 0.0105 to 0.1039)

Study | ES [95% Conf. Interval]

---------------------+---------------------------------------------------

Mitrakul (1984) | 0.0000 0.0000 0.0755

Wongtongkam (2005) | 0.0235 0.0029 0.0824

Wongtongkam (2005) | 0.0578 0.0311 0.0968

---------------------+---------------------------------------------------

Random pooled ES | 0.0339 0.0105 0.1039

---------------------+---------------------------------------------------

LR test: RE vs FE Model chi^2 = 0.2354 (d.f. = 1) p = 0.3138

Estimate of between-study variance Tau^2 = 0.1679

Test of ES=0 : z= -5.4922 p = 0.0000

- Number of snakebite victims who sought traditional healer first then switched to conventional treatment in 2019 = 0.0339 x 8,704 = 295 cases

- Number of snakebite victims who sought conventional treatment only = 8,704 – 295 = 8,409

- Proportion of snakebite victims who sought conventional treatment only = $\frac{8,409}{8,715}$ = 0.965

- Proportion of snakebite victims who sought traditional healer first = 1 – 0.965 = 0.035

- Proportion of snakebite victims who sought traditional healer first then switched to conventional treatment = $\frac{295}{305}$ = 0.034

- Proportion of snakebite victims who sought only traditional healer = 1 – 0.034 = 0.966

**Mortality of snakebite**

- Probability of death of systemic envenoming treated with antivenom in healthcare facilities in 2014 to 2018[28] = $\frac{20}{25,747}$ = 0.0008 (95%CI 0.0005 to 0.0012)

- Relative risk of death in snakebite envenoming without antivenom treatment compared to with antivenom treatment[6] = 2.33

- Probability of death of systemic envenoming treated without antivenom treatment in healthcare facilities = 0.0008 x 2.33 = 0.0018

- Probability of death of systemic envenoming not treated in healthcare facilities = 0.0012 x 2.33 = 0.0028

**Amputation following snakebite envenoming**

- Probability of bitten by *Naja kaouthia* in 2019[12] = $\frac{578}{5,160}$ = 0.112

- Probability of digit amputation following *Naja kaouthia* bite derived from meta-analysis[13, 16, 20, 22, 25, 27, 29-32] = 0.003 (95%CI 0.001 to 0.012)

Study | ES [95% Conf. Interval]

------------------------+---------------------------------------------------

Trishnananda (1979) | 0.0000 0.0000 0.1684

Mitrakul (1984) | 0.0000 0.0000 0.2180

Malasit (1986) | 0.0000 0.0000 0.0451

Looareesuwan (1988) | 0.0000 0.0000 0.0771

Viravan (1992) | 0.0000 0.0000 0.0435

Buranasin (1993) | 0.0000 0.0000 0.0974

Pochanugool (1998) | 0.0000 0.0000 0.6024

Dumavibhat (1997) | 0.0000 0.0000 0.0672

Pochanugool (1997) | 0.0000 0.0000 0.0528

Pochanugool (1997) | 0.0000 0.0000 0.0430

Wongtongkam (2005) | 0.0118 0.0003 0.0638

Thiansookon (2008) | 0.0000 0.0000 0.1089

Laohawiriyakamol (2011) | 0.0172 0.0004 0.0924

------------------------+---------------------------------------------------

Random pooled ES | 0.0030 0.0008 0.0120

------------------------+---------------------------------------------------

LR test: RE vs FE Model chi^2 = 0.0000 (d.f. = 11) p = .

Estimate of between-study variance Tau^2 = 0.0000

Test of ES=0 : z= -8.1931 p = 0.0000

- Probability of digit amputation following snakebite = 0.112 x 0.003 = 0.0003 (95%CI 0.0001 to 0.0013)

- Probability of limb amputation following snakebite = 0.00

**Hospitalization costs for snakebite victims with systemic envenoming**

- Hospitalization costs comprise of inpatient department services, laboratory, tetanus toxoid, and wound dressing.

- Length of stay of victims hospitalized for systemic envenoming derived from meta-analysis[15, 19, 23, 24, 27, 29, 31] = 3.5 days (95%CI 2.6 to 4.4)

Meta-analysis summary Number of studies = 14

Random-effects model Heterogeneity:

Method: REML tau2 = 1.7677

I2 (%) = 94.09

H2 = 16.92

--------------------------------------------------------------------

Study | Effect Size [95% Conf. Interval] % Weight

------------------+-------------------------------------------------

Mitrakul (1991) | 1.500 1.108 1.892 11.11

Buranasin (1993) | 2.300 1.849 2.751 11.03

Buranasin (1993) | 2.000 -2.371 6.371 2.98

Pochanugool (1997)| 10.090 1.976 18.204 1.06

Wongtongkam (2005)| 5.500 -0.713 11.713 1.70

Wongtongkam (2005)| 5.650 3.925 7.375 7.90

Wongtongkam (2005)| 2.870 1.870 3.870 9.91

Wongtongkam (2005)| 5.340 3.596 7.084 7.85

Wongtongkam (2005)| 3.020 -0.371 6.411 4.22

Wongtongkam (2005)| 4.350 3.076 5.624 9.17

Wongtongkam (2005)| 2.150 1.895 2.405 11.26

Wongtongkam (2005)| 4.330 2.154 6.506 6.70

Tongpoo (2018) | 7.000 3.394 10.606 3.90

Thumtecho (2020) | 3.000 2.706 3.294 11.22

------------------+-------------------------------------------------

theta | 3.498 2.620 4.377

--------------------------------------------------------------------

Sorted by: _meta_id

Test of theta = 0: z = 7.81 Prob > |z| = 0.0000

Test of homogeneity: Q = chi2(13) = 88.18 Prob > Q = 0.0000

| **Item** | **Quantity** | **Price (THB)** | **Cost (THB)** |
| --- | --- | --- | --- |
| **Hospitalization costs, total** |  |  | **9,992.25** |
| **Inpatient department services** | **3.5** | 1,948.57[33] | **6,816.09** |
| **Laboratory for systemic envenoming, average** |  |  | **2,385.66** |
| - Venous clotting time | 7^*^ | 59.64[33] | 417.49 |
| - Prothrombin time | 12^*^ | 90.05[33] | 1,080.56 |
| - Complete blood count | 7^*^ | 59.64[33] | 417.49 |
| - Urine analysis | 1^*^ | 74.84[33] | 74.84 |
| - Electrolyte | 1^*^ | 120.45[33] | 120.45 |
| - Blood urea nitrogen | 1^*^ | 74.84[33] | 43.98 |
| - Creatinine | 1^*^ | 74.84[33] | 74.84 |
| **Tetanus toxoid** |  |  | **54.11** |
| - Tetanus toxoid | 1^*^ | 23.71[33] | 23.54 |
| - Intramuscular drug administration | 1^*^ | 30.41[33] | 30.41 |
| **Wound dressing** | **3.5** | **210.52**[33] | **736.39** |

Note: * – expert opinion; THB – Thai Baht where 31.05 THB = 1 United States Dollar.

**Hospitalization costs for victims without snakebite envenoming**

- Hospitalization costs comprise of inpatient department services, laboratory, tetanus toxoid, and wound dressing.

| **Item** | **Quantity** | **Price (THB)** | **Cost (THB)** |
| --- | --- | --- | --- |
| **Hospitalization costs, total** |  |  | **3,209.23** |
| **Inpatient department services** | **1** | 2,578.90[33] | **2,578.90** |
| **Laboratory for snakebite without systemic envenoming** |  |  | **630.33** |
| - Venous clotting time | 1^*^ | 59.64[33] | 59.64 |
| - Prothrombin time | 1^*^ | 90.05[33] | 90.05 |
| - Complete blood count | 1^*^ | 59.64[33] | 59.64 |
| - Urine analysis | 1^*^ | 74.84[33] | 74.84 |
| - Electrolyte | 1^*^ | 120.45[33] | 120.45 |
| - Blood urea nitrogen | 1^*^ | 74.84[33] | 43.98 |
| - Creatinine | 1^*^ | 74.84[33] | 74.84 |
| **Tetanus toxoid** |  |  | **54.11** |
| - Tetanus toxoid | 1^*^ | 23.71[33] | 23.54 |
| - Intramuscular drug administration | 1^*^ | 30.41[33] | 30.41 |
| **Wound dressing** | **1**^*^ | **210.52**[33] | **210.52** |

Note: * – expert opinion; THB – Thai Baht where 31.05 THB = 1 United States Dollar.

**Antivenom treatment costs**

- Antivenom treatment costs comprise of antivenom, and antivenom administration.

| **Item** | **Quantity** | **Price (THB)** | **Cost (THB)** |
| --- | --- | --- | --- |
| **Antivenom treatment costs, total** |  |  | **6,996.59** |
| **Antivenom, average** |  |  | **6,748.57** |
| Proportion of snakes[12] |  |  |  |
| Antivenom, hematotoxic snakes | 6^*^ | 1,000 to 1,200* | 6,000 to 7,200 |
| Antivenom, neurotoxic snakes | 5 to 10^*^ | 1,000 to 1,200* | 5,000 to 12,000 |
| **Antivenom administration** |  |  | **248.03** |
| - IV set | 1^*^ | 150.86[33] | 150.86 |
| - IV drug administration | 2^*^ | 30.41[33] | 60.81 |
| - 0.9% NaCl 100 mL | 2^*^ | 18.18[33] | 36.36 |

Note: * – expert opinion; THB – Thai Baht where 31.05 THB = 1 United States Dollar.

**Adverse reaction management costs**

| **Item** | **Quantity** | **Price (THB)** | **Cost (THB)** |
| --- | --- | --- | --- |
| **Adverse reaction management costs, total** |  |  | **105.46** |
| - Chlorpheniramine | 1^*^ | 2.27[33] | 2.27 |
| - Adrenaline | 1^*^ | 5.72[33] | 5.72 |
| - Dexamethasone | 1^*^ | 6.25[33] | 6.25 |
| - IV drug administration | 3^*^ | 30.41[33] | 91.22 |

Note: * – expert opinion; THB – Thai Baht where 31.05 THB = 1 United States Dollar.

**Indonesia**

**Incidence of snakebite**
- Total number of snakebite victims in 2019 (expert opinion) = 135,000 cases

- Number of population in 2019[3] = 270,625,568 people
- Incidence of snakebite in 2019 = $\frac{135,000}{270,625,568}$ = 49.88 per 100,000 population per year

**Treatment seeking behavior**

- Proportion of snakebite victims treated in healthcare facilities (expert opinion) = 0.75

- Proportion of snakebite victims who sought only traditional treatment (expert opinion) = 1-0.75 = 0.25

- Proportion of snakebite victims in healthcare facilities who sought traditional healer first then switched to conventional treatment (expert opinion) = 0.00

**Mortality of snakebite**

- Proportion of snakebite envenoming treated with antivenom from 2004 to 2009[34] = $\frac{17}{42}$ = 0.40 (95%CI 0.26 to 0.57)

- Probability of death of systemic envenoming treated with antivenom treatment in healthcare facilities in 2019 (local data) = $\frac{54}{587}$ = 0.09 (95%CI 0.07 to 0.12)

- Relative risk of death in snakebite envenoming without antivenom treatment compared to with antivenom treatment[6]= 2.33

- Probability of death of systemic envenoming treated without antivenom treatment in healthcare facilities = 0.09 x 2.33 = 0.21

- Probability of death of systemic envenoming not treated in healthcare facilities = 0.12 x 2.33 = 0.28

**Amputation due to snakebite envenoming**

**-** Probability of amputation due to snakebite envenoming in 2019 (local data) = $\frac{12}{587}$ = 0.02 (95%CI 0.01 to 0.04)

- Proportion of digit amputation to limb amputation (expert opinion) = 0.50
**-** Probability of limb amputation due to snakebite envenoming in 2019 (local data) = 0.02 x 0.50 = 0.01 (95%CI 0.005 to 0.018)

**Hospitalization costs for snakebite victims with systemic envenoming**

- Hospitalization costs comprise of inpatient department services, laboratory, and tetanus toxoid.

| **Item** | **Quantity** | **Price (IDR)** | **Cost (IDR)** |
| --- | --- | --- | --- |
| **Hospitalization costs, total** |  |  | **14,253,228** |
| **Inpatient department services including laboratory based on diagnosis group of non-infectious bacteria for region 1 / class 3 / secondary hospital** | **6.1[5]** | **2,326,565*** | **14,103,637** |
| **Tetanus toxoid** |  |  | **149,591** |
| - Tetanus toxoid | 1^*^ | 146,800[35] | 146,800 |
| - Needle | 1^*^ | 734[9] | 734 |
| - Syringe | 1^*^ | 2,056[9] | 2,056 |

Note: * – expert opinion; IDR – Indonesian Rupee where 14,147.67 IDR = 1 United States Dollar.

**Hospitalization costs for victims without snakebite envenoming**

- Hospitalization costs comprise of inpatient department services, laboratory, and tetanus toxoid.

| **Item** | **Quantity** | **Price (IDR)** | **Cost (IDR)** |
| --- | --- | --- | --- |
| **Hospitalization costs, total** |  |  | **2,476,156** |
| **Inpatient department services including laboratory based on diagnosis group of non-infectious bacteria for region 1 / class 3 / secondary hospital** | **1** | **2,326,565*** | **2,326,565** |
| **Tetanus toxoid** |  |  | **149,591** |
| - Tetanus toxoid | 1^*^ | 146,800[35] | 146,800 |
| - Needle | 1^*^ | 734[9] | 734 |
| - Syringe | 1^*^ | 2,056[9] | 2,056 |

Note: * – expert opinion; IDR – Indonesian Rupees where 14,147.67 IDR = 1 United States Dollar.

**Antivenom treatment costs**

- Antivenom treatment costs comprise of antivenom, and antivenom administration.

| **Item** | **Quantity** | **Price (IDR)** | **Cost (IDR)** |
| --- | --- | --- | --- |
| **Antivenom treatment costs, total** |  |  | **10,931,952** |
| **Antivenom**[35] |  |  | **10,892,732** |
| **Antivenom administration** |  |  | **39,220** |
| - Needle | 1^*^ | 734[9] | 734 |
| - Syringe | 1^*^ | 2,056[9] | 2,056 |
| - 0.9% NaCl 100 mL | 1^*^ | 7,197[9] | 7,197 |
| - IV set | 1^*^ | 36,429[9] | 36,429 |

Note: * – expert opinion; IDR – Indonesian Rupee where 14,147.67 IDR = 1 United States Dollar.

**Adverse reaction management costs**

| **Item** | **Quantity** | **Price (IDR)** | **Cost (IDR)** |
| --- | --- | --- | --- |
| **Adverse reaction management costs, total** |  |  | **54,373** |
| - Chlorpheniramine | 1^*^ | 14,000[35] | 14,000 |
| - Adrenaline | 1^*^ | 18,000[35] | 18,000 |
| - Dexamethasone | 1^*^ | 14,000[35] | 14,000 |
| - Needle | 3^*^ | 734[9] | 2,203 |
| - Syringe | 3^*^ | 2,056[9] | 6,169 |

Note: * – expert opinion; IDR – Indonesian Rupee where 14,147.67 IDR = 1 United States Dollar.

**Philippines**

**Treatment seeking behavior**

- Proportion of snakebite victims seeking only conventional treatment (expert opinion) = 0.00

- Proportion of snakebite victims treated in healthcare facilities in 1987[36] = $\frac{2}{24}$ =0.083 (95%CI 0.10 to 0.27) then applied higher estimate 0.27 for base-case analysis

- Proportion of snakebite victims in healthcare facilities who sought traditional healer first then switched to conventional treatment[36] = 0.27

- Proportion of snakebite victims who sought only traditional treatment = 1-0.27 = 0.73

**Incidence of snakebite**
- Number of snakebite patients treated in healthcare facility in 2019 (local data) = 157 per 4,700,000 population
- Incidence of snakebite patients treated in healthcare facilities in 2019 = $\frac{157}{4,700,000}$ = 3.34 per 100,000 population per year

- Number of population in 2019[3] = 108,116,615 people
- Number of snakebite patients treated in healthcare facilities in 2019 = $\frac{3.34}{100,000}$ x 108,116,615 = 3,612 cases

- Total number of snakebite victims = $\frac{3,612}{0.27}$ x 108,116,615 = 13,377 cases

- Incidence of snakebite in 2019 = $\frac{13,377}{108,111,615}$ = 12.37 per 100,000 population per year

**Mortality of snakebite**

- Proportion of snakebite envenoming treated with antivenom from 2018 to 2019 (local data) = $\frac{45}{279}$ = 0.1613

- Probability of death of systemic envenoming treated with antivenom treatment in healthcare facilities from 2018 to 2019 (local data) = $\frac{3}{45}$ = 0.067 (95%CI 0.014 to 0.183)

- Relative risk of death in snakebite envenoming without antivenom treatment compared to with antivenom treatment[6]= 2.33

- Probability of death of systemic envenoming treated without antivenom treatment in healthcare facilities = 0.067 x 2.33 = 0155

- Probability of death of systemic envenoming not treated in healthcare facilities = 0.12 x 2.33 = 0.426

**Amputation due to snakebite envenoming**

**-** Probability of amputation due to snakebite envenoming (expert opinion) = 0.01

- Proportion of digit amputation to limb amputation (expert opinion) = 0.70:0.30

**-** Probability of digit amputation due to snakebite envenoming = 0.01 x 0.70 = 0.007
**-** Probability of limb amputation due to snakebite envenoming = 0.01 x 0.30 = 0.003

**Hospitalization costs for snakebite victims with systemic envenoming**

- Hospitalization costs comprise of inpatient department services, laboratory, and tetanus toxoid.

| **Item** | **Quantity** | **Price (PHP)** | **Cost (PHP)** |
| --- | --- | --- | --- |
| **Hospitalization costs, total** |  |  | **21,135** |
| **Inpatient department services including laboratory** | **6.1[5]** | **3,479[37]** | **21,088** |
| **Tetanus toxoid** |  |  | **47** |
| - Tetanus toxoid | 1^*^ | 36[38] | 36 |
| - Needle | 1^*^ | 1[38] | 1 |
| - Syringe | 1^*^ | 10[38] | 10 |

Note: * – expert opinion; PHP – Philippines Peso where 51.80 PHP = 1 United States Dollar.

**Hospitalization costs for victims without snakebite envenoming**

- Hospitalization costs comprise of inpatient department services, laboratory, and tetanus toxoid.

| **Item** | **Quantity** | **Price (PHP)** | **Cost (PHP)** |
| --- | --- | --- | --- |
| **Hospitalization costs, total** |  |  | **3,526** |
| **Inpatient department services including laboratory** | **1** | **3,479[37]** | **3,479** |
| **Tetanus toxoid** |  |  | **47** |
| - Tetanus toxoid | 1^*^ | 36[38] | 36 |
| - Needle | 1^*^ | 1[38] | 1 |
| - Syringe | 1^*^ | 10[38] | 10 |

Note: * – expert opinion; PHP – Philippines Peso where 51.80 PHP = 1 United States Dollar.

**Antivenom treatment costs**

- Antivenom treatment costs comprise of antivenom, and antivenom administration.

| **Item** | **Quantity** | **Price (PHP)** | **Cost (PHP)** |
| --- | --- | --- | --- |
| **Antivenom treatment costs, total** |  |  | **16,161** |
| **Antivenom** | 10* | **1,600*** | **16,000** |
| **Antivenom administration** |  |  | **161** |
| - Needle | 1^*^ | 1[38] | 1 |
| - Syringe | 1^*^ | 10[38] | 10 |
| - 0.9% NaCl 100 mL | 2^*^ | 60[38] | 120 |
|  | 2^*^ | 60[38] | 120 |
| - IV set | 1^*^ | 30[38] | 30 |

Note: * – expert opinion; PHP – Philippines Peso where 51.80 PHP = 1 United States Dollar.

**Adverse reaction management costs**

| **Item** | **Quantity** | **Price (PHP)** | **Cost (PHP)** |
| --- | --- | --- | --- |
| **Adverse reaction management costs, total** |  |  | **230** |
| - Diphenhydramine | 1^*^ | 23[38] | 23 |
| - Adrenaline | 1^*^ | 24[38] | 24 |
| - Hydrocortisone | 1^*^ | 150[38] | 150 |
| - Needle | 3^*^ | 1[38] | 4 |
| - Syringe | 3^*^ | 10[38] | 29 |

Note: * – expert opinion; PHP – Philippines Peso where 51.80 PHP = 1 United States Dollar.

**Vietnam**

**Incidence of snakebite**
- Incidence of snakebite patients treated in healthcare facilities in 2017[39] = 20.98 cases per 100,000 population
- Total incidence of snakebite victims in 2017[39] = 48.46 cases per 100,000 population
- Proportion of snakebite victims treated in healthcare facilities in 2017[39]= $\frac{20.98}{48.46}$ =0.43 (95%CI 0.29 to 0.59)

**Treatment seeking behavior**

- Proportion of snakebite victims seeking only conventional treatment (expert opinion) = 0.00

- Proportion of snakebite victims in healthcare facilities who sought traditional healer first then switched to conventional treatment[39] = 0.43

- Proportion of snakebite victims who sought only traditional treatment = 1-0.43 = 0.57

**Mortality of snakebite**

- Probability of death of systemic envenoming treated with antivenom in healthcare facilities (local data) = $\frac{15}{1,000}$ = 0.015 (95%CI 0.008 to 0.025)

- Relative risk of death in snakebite envenoming without antivenom treatment compared to with antivenom treatment[6]= 2.33

- Probability of death of systemic envenoming treated without antivenom treatment in healthcare facilities = 0.015 x 2.33 = 0.035

- Probability of death of systemic envenoming not treated in healthcare facilities = 0.025 x 2.33 = 0.057

**Hospitalization costs for snakebite victims with systemic envenoming**

- Hospitalization costs comprise of inpatient department services, laboratory, and tetanus toxoid.

| **Item** | **Quantity** | **Price (VND)** | **Cost (VND)** |
| --- | --- | --- | --- |
| **Hospitalization costs, total** |  |  | **3,922,215** |
| **Inpatient department services including laboratory** | **6.1[5]** | **647,017[40]** | **3,981,731** |
| **Tetanus toxoid** |  |  | **59,515** |
| - Tetanus toxoid | 1^*^ | 42,049^*^ | 42,049 |
| - Needle | 1^*^ | 2,417^*^ | 2,417 |
| - Syringe | 1^*^ | 15,049^*^ | 15,049 |

Note: * – expert opinion; VND – Vietnamese Dong where 23,050.24 VND = 1 United States Dollar.

**Hospitalization costs for victims without snakebite envenoming**

- Hospitalization costs comprise of inpatient department services, laboratory, and tetanus toxoid.

| **Item** | **Quantity** | **Price (VND)** | **Cost (VND)** |
| --- | --- | --- | --- |
| **Hospitalization costs, total** |  |  | **706,532** |
| **Inpatient department services including laboratory** | 1^*^ | **647,017[40]** | **647,017** |
| **Tetanus toxoid** |  |  | **59,515** |
| - Tetanus toxoid | 1^*^ | 42,049^*^ | 42,049 |
| - Needle | 1^*^ | 2,417^*^ | 2,417 |
| - Syringe | 1^*^ | 15,049^*^ | 15,049 |

Note: * – expert opinion; VND – Vietnamese Dong where 23,050.24 VND = 1 United States Dollar.

**Antivenom treatment costs**

- Antivenom treatment costs comprise of antivenom, and antivenom administration.

| **Item** | **Quantity** | **Price (VND)** | **Cost (VND)** |
| --- | --- | --- | --- |
| **Antivenom treatment costs, total** |  |  | **1,565,316** |
| **Antivenom, average*** |  |  | **1,491,486** |
| **Antivenom administration** |  |  | **73,830** |
| - Needle | 1^*^ | 2,417^*^ | 2,417 |
| - Syringe | 1^*^ | 15,049^*^ | 15,049 |
| - 0.9% NaCl 100 mL | 2^*^ | 23,370^*^ | 46,741 |
| - IV set | 1^*^ | 27,089 ^*^ | 27,089 |

Note: * – expert opinion; VND – Vietnamese Dong where 23,050.24 VND = 1 United States Dollar.

**Adverse reaction management costs**

| **Item** | **Quantity** | **Price (VND)** | **Cost (VND)** |
| --- | --- | --- | --- |
| **Adverse reaction management costs, total** |  |  | **118,703** |
| - Chlorpheniramine | 1^*^ | 24,878^*^ | 24,878 |
| - Adrenaline | 1^*^ | 23,813^*^ | 23,813 |
| - Dexamethasone | 1^*^ | 17,705^*^ | 17,705 |
| - Needle | 3^*^ | 2,417^*^ | 7250 |
| - Syringe | 3^*^ | 15,049^*^ | **45,148** |

Note: * – expert opinion; VND – Vietnamese Dong where 23,050.24 VND = 1 United States Dollar.

**Lao PDR**

**Incidence of snakebite**
- Total incidence of snakebite in 2019 (expert opinion) = 200.00 per 100,000 population per year
- Number of population in 2019[3] = 7,169,455 people
- Total number of snakebite victims = $\frac{200.00}{100,000}$ x 7,169,455 = 14,339 cases

**Treatment seeking behavior**

- Proportion of snakebite victims seeking only conventional treatment (expert opinion) = 0.00

- Proportion of snakebite victims treated in healthcare facilities (expert opinion) = 0.10

- Proportion of snakebite victims in healthcare facilities who sought traditional healer first then switched to conventional treatment = 0.10

- Proportion of snakebite victims who sought only traditional treatment = 1-0.10 = 0.90

**Mortality of snakebite**

- Probability of systemic envenoming needed antivenom treatment in 2014[41] = $\frac{43}{158}$ = 0.27 (95%CI 0.20 to 0.35)

- Probability of death of systemic envenoming treated in healthcare facilities in 2014[41] = $\frac{2}{43}$ = 0.05 (95%CI 0.01 to 0.16)

- Relative risk of death in snakebite envenoming without antivenom treatment compared to with antivenom treatment[6]= 2.33

- Probability of death of systemic envenoming treated without antivenom treatment in healthcare facilities = 0.05 x 2.33 = 0.11

- Probability of death of systemic envenoming not treated in healthcare facilities = 0.16 x 2.33 = 0.37

**Amputation due to snakebite envenoming**

**-** Probability of digit amputation due to snakebite envenoming in 2014[41] = $\frac{2}{43}$ = 0.05 (95%CI 0.01 to 0.16)

- Probability of limb amputation due to snakebite envenoming in 2014[41] = $\frac{1}{43}$ = 0.02 (95%CI 0.001 to 0.12)

**Hospitalization costs for snakebite victims with systemic envenoming**

- Hospitalization costs comprise of inpatient department services, laboratory, tetanus toxoid, and wound dressing.

| **Item** | **Quantity** | **Price (LAK)** | **Cost (LAK)** |
| --- | --- | --- | --- |
| **Hospitalization costs, total** |  |  | **3,233,043** |
| **Inpatient department services** | **6.1[5]** | **90,000^*^** | **545,580** |
| **Laboratory for systemic envenoming, average** |  |  | **2,301,333** |
| - Coagulation profile | 7^*^ | 52,667^*^ | 368,667 |
| - Complete blood count | 4^*^ | 43,667^*^ | 174,667 |
| - Urine analysis | 6^*^ | 61,000^*^ | 366,000 |
| - Electrolyte | 6^*^ | 96,000^*^ | 576,000 |
| - Blood urea nitrogen | 6^*^ | 48,000^*^ | 288,000 |
| - Creatinine | 6^*^ | 44,000^*^ | 264,000 |
| - Creatine kinase | 6^*^ | 44,000^*^ | 264,000 |
| **Tetanus toxoid** |  |  | **22,410** |
| - Tetanus toxoid | 1^*^ | 15,833^*^ | 15,833 |
| - Needle | 1^*^ | 910^*^ | 910 |
| - Syringe | 1^*^ | 5,667^*^ | 5,667 |
| **Wound dressing** | **6.1[5]** | **60,000^*^** | **363,720** |

Note: * – expert opinion; LAK – Lao Kip where 8,679.41 LAK = 1 United States Dollar.

**Hospitalization costs for victims without snakebite envenoming**

- Hospitalization costs comprise of inpatient department services, laboratory, tetanus toxoid, and wound dressing.

| **Item** | **Quantity** | **Price (LAK)** | **Cost (LAK)** |
| --- | --- | --- | --- |
| **Hospitalization costs, total** |  |  | **598,077** |
| **Inpatient department services** | 1^*^ | **90,000^*^** | **90,000** |
| **Laboratory for snakebite without systemic envenoming** |  |  | **485,667** |
| - Coagulation profile | 2^*^ | 52,667^*^ | 105,333 |
| - Complete blood count | 2^*^ | 43,667^*^ | 87,333 |
| - Urine analysis | 1^*^ | 61,000^*^ | 61,000 |
| - Electrolyte | 1^*^ | 96,000^*^ | 96,000 |
| - Blood urea nitrogen | 1^*^ | 48,000^*^ | 48,000 |
| - Creatinine | 1^*^ | 44,000^*^ | 44,000 |
| - Creatine kinase | 1^*^ | 44,000^*^ | 44,000 |
| **Tetanus toxoid** |  |  | **22,410** |
| - Tetanus toxoid | 1^*^ | 15,833^*^ | 15,833 |
| - Needle | 1^*^ | 910^*^ | 910 |
| - Syringe | 1^*^ | 5,667^*^ | 5,667 |
| **Wound dressing** | 1^*^ | **60,000^*^** | **60,000** |

Note: * – expert opinion; LAK – Lao Kip where 8,679.41 LAK = 1 United States Dollar.

**Antivenom treatment costs**

- Antivenom treatment costs comprise of antivenom, and antivenom administration.

| **Item** | **Quantity** | **Price (LAK)** | **Cost (LAK)** |
| --- | --- | --- | --- |
| **Antivenom treatment costs, total** |  |  | **1,715,377** |
| **Antivenom, average** | **3^*^** | **560,334^*^** | **1,681,000** |
| **Antivenom administration** |  |  | **34,376** |
| - Needle | 1^*^ | 910^*^ | 910 |
| - Syringe | 1^*^ | 5,667^*^ | 5,667 |
| - 0.9% NaCl 100 mL | 2^*^ | 8,800^*^ | 17,600 |
| - IV set | 1^*^ | 10,200^*^ | 10,200 |

Note: * – expert opinion; LAK – Lao Kip where 8,679.41 LAK = 1 United States Dollar.

**Adverse reaction management costs**

| **Item** | **Quantity** | **Price (LAK)** | **Cost (LAK)** |
| --- | --- | --- | --- |
| **Adverse reaction management costs, total** |  |  | **44,697** |
| - Diphenhydramine | 1^*^ | 9,333^*^ | 9,333 |
| - Adrenaline | 1^*^ | 8,967^*^ | 8,967 |
| - Dexamethasone | 1^*^ | 6,667^*^ | 6,667 |
| - Needle | 3^*^ | 910^*^ | 2,730 |
| - Syringe | 3^*^ | 5,667^*^ | 17,000 |

Note: * – expert opinion; LAK – Lao Kip where 8,679.41 LAK = 1 United States Dollar.

**Myanmar**

**Treatment seeking behavior**
- Incidence of snakebite victims reported in communities in 2015[42] = 116 cases per 100,000 population (95%CI 74 to 182)

- Incidence of snakebite patients treated in healthcare facilities in 2015[42] = 44 cases per 100,000 population
- Proportion of snakebite patients treated in healthcare facilities in 2015[42] = $\frac{44}{116}$ = 0.38

- Proportion of snakebite victims treated in healthcare facilities who have sought traditional treatment before in 2015[42] = $\frac{111}{965}$ = 0.12 (95%CI 0.10 to 0.14)

- Number of snakebite patients seeking traditional treatment only in 2015[42] = $\frac{965}{0.38}$ = 2,544 cases

- Proportion of snakebite victims seeking only conventional treatment in 2015[42] = $\frac{854}{2,544}$ = 0.34

- Proportion of snakebite victims in healthcare facilities who sought traditional healer first then switched to conventional treatment in 2015[42] = $\frac{111}{1,690}$ = 0.07

- Proportion of snakebite victims who sought only traditional treatment in 2015[42] = 1-0.07 = 0.93

**Incidence of snakebite**
- Number of snakebite patients treated in healthcare facilities in 2019 (data from the Ministry of Health and Sports Myanmar) = 7,988 cases

- Total number of snakebite victims = $\frac{7,988}{0.38}$ = 21,059 cases
- Number of population in 2019[3] = 54,045,420 people
- Incidence of snakebite in 2019 = $\frac{21,059}{54,045,420}$ = 38.97 per 100,000 population per year

**Mortality of snakebite**

**-** Proportion of snakebite envenoming treated with antivenom in healthcare facilities 2015[42] = $\frac{762}{965}$ = 0.79 (95%CI 0.76 to 0.81)

- Number of snakebite envenoming treated with antivenom in healthcare facilities 2015[42] = 7,988 x 0.79 = 6,308 cases

- Number of deaths from snakebite envenoming treated in healthcare facilities in 2019 (data from the Ministry of Health and Sports Myanmar) = 426 deaths

- Probability of death of systemic envenoming treated with antivenom in healthcare facilities in 2019 = $\frac{426}{6,308}$ = 0.068 (95%CI 0.061 to 0.074)

- Relative risk of death in snakebite envenoming without antivenom treatment compared to with antivenom treatment[6]= 2.33

- Probability of death of systemic envenoming treated without antivenom treatment in healthcare facilities = 0.068 x 2.33 = 0.157

- Probability of death of systemic envenoming not treated in healthcare facilities = 0.074 x 2.33 = 0.172

**Hospitalization costs for snakebite victims with systemic envenoming**

- Hospitalization costs comprise of inpatient department services, laboratory, and tetanus toxoid.

| **Item** | **Quantity** | **Price (MMK)** | **Cost (MMK)** |
| --- | --- | --- | --- |
| **Hospitalization costs, total** |  |  | **318,232** |
| **Inpatient department services including laboratory** | **6.1[5]** | **52,163[43]** | **316,212** |
| **Tetanus toxoid** |  |  | **2,020** |
| - Tetanus toxoid | 1^*^ | 1,900^*^ | 1,900 |
| - Needle | 1^*^ | 20^*^ | 20 |
| - Syringe | 1^*^ | 200^*^ | 200 |

Note: * – expert opinion; MMK – Myanmar Kyat where 1,518.26 MMK = 1 United States Dollar.

**Hospitalization costs for victims without snakebite envenoming**

- Hospitalization costs comprise of inpatient department services, laboratory, and tetanus toxoid.

| **Item** | **Quantity** | **Price (MMK)** | **Cost (MMK)** |
| --- | --- | --- | --- |
| **Hospitalization costs, total** |  |  | **54,183** |
| **Inpatient department services including laboratory** | 1^*^ | **52,163[43]** | **52,163** |
| **Tetanus toxoid** |  |  | **2,020** |
| - Tetanus toxoid | 1^*^ | 1,900^*^ | 1,900 |
| - Needle | 1^*^ | 20^*^ | 20 |
| - Syringe | 1^*^ | 100^*^ | 100 |

Note: * – expert opinion; MMK – Myanmar Kyat where 1,518.26 MMK = 1 United States Dollar.

**Antivenom treatment costs**

- Antivenom treatment costs comprise of antivenom, and antivenom administration.

| **Item** | **Quantity** | **Price (MMK)** | **Cost (MMK)** |
| --- | --- | --- | --- |
| **Antivenom treatment costs, total** |  |  | **494,883** |
| **Antivenom, average*** |  |  | **494,463** |
| **Antivenom administration** |  |  | **420** |
| - Needle | 1^*^ | 20^*^ | 20 |
| - Syringe | 1^*^ | 100^*^ | 100 |
| - IV set | 1^*^ | 300 ^*^ | 300 |

Note: * – expert opinion; MMK – Myanmar Kyat where 1,518.26 MMK = 1 United States Dollar.

**Adverse reaction management costs**

| **Item** | **Quantity** | **Price (MMK)** | **Cost (MMK)** |
| --- | --- | --- | --- |
| **Adverse reaction management costs, total** |  |  | **1,160** |
| - Chlorpheniramine | 1^*^ | 200^*^ | 200 |
| - Adrenaline | 1^*^ | 350^*^ | 350 |
| - Dexamethasone | 1^*^ | 250^*^ | 250 |
| - Needle | 3^*^ | 20^*^ | 60 |
| - Syringe | 3^*^ | 100^*^ | 300 |

Note: * – expert opinion; MMK – Myanmar Kyat where 1,518.26 MMK = 1 United States Dollar.

**References**

1. Kasturiratne A, Wickremasinghe AR, de Silva N, Gunawardena NK, Pathmeswaran A, Premaratna R, et al. The global burden of snakebite: a literature analysis and modelling based on regional estimates of envenoming and deaths. PLoS Med. 2008;5(11):e218.

2. Sivaganabalan R, Ismail AK, Salleh MS, Mohan K, Tan CH, Adnan A. Guideline on the Management of Snakebites. Ministry of Health Malaysia; 2017.

3. World Bank. Population, total [Internet]. 2019. Available from: <https://data.worldbank.org/indicator/SP.POP.TOTL>.

4. Longbottom J, Shearer FM, Devine M, Alcoba G, Chappuis F, Weiss DJ, et al. Vulnerability to snakebite envenoming: A global mapping of hotspots. The Lancet. 2018;392(10148):673-84.

5. Shafie NA, Fauzi H, Wahab M, Senek M, Ismail A. The prevalence of hypersensitivity reactions to snake antivenoms administered in sultanah nur zahirah hospital from 2013 to 2016. Med J Malaysia. 2020;75(3):217.

6. Habib AG, Warrell DA. Antivenom therapy of carpet viper (Echis ocellatus) envenoming: effectiveness and strategies for delivery in West Africa. Toxicon. 2013;69:82-9.

7. Attorney General’s Chambers Malaysia. Fees (Medical) (Cost of Services) Order 2014. Federal Government Gazette; 2014.

8. Drugs and Medical Supplies Information Center (DMSIC), Ministry of Public Health Thailand. Reference drug price [Internet]. Available from: <http://dmsic.moph.go.th/price/price1_1.php?method=drug>.

9. Chanthawong S, Lim YH, Subongkot S, Chan A, Andalusia R, Bustamam RSA, et al. Cost-effectiveness analysis of olanzapine-containing antiemetic therapy for managing highly emetogenic chemotherapy in Southeast Asia: a multinational study. Supportive Care in Cancer. 2019;27(3):1109-19.

10. Lam A, Zaim M, Helmy H, Ramdhan I. Economic Impact of Managing Acute Diabetic Foot Infection in a Tertiary Hospital in Malaysia. Malaysian Orthopaedic Journal. 2014;8(1):46.

11. Pharmaceutical Services Programme, Ministry of Health Malaysia,. Consumer Price Guide [Internet]. Available from: <https://www.pharmacy.gov.my/v2/en/apps/drug-price>.

12. National Health Security Office, Thailand. Report on the creation of the National Health Security for fiscal year BE 2562 (AD 2019). Bangkok, Thailand; 2019.

13. Mitrakul C, Dhamkrong-At A, Futrakul P, Thisyakorn C, Vongsrisart K, Varavithya C, et al. Clinical features of neurotoxic snake bite and response to antivenom in 47 children. Am J Trop Med Hyg. 1984;33(6):1258-66. Epub 1984/11/01. doi: 10.4269/ajtmh.1984.33.1258. PubMed PMID: 6507733.

14. Hutton RA, Looareesuwanzj S, Ho M, Silamut K, Chanthavanich P, Karbwang J, et al. Arboreal green pit vipers (genus Trimeresurus) of south-east Asia: Bites by T. albolabris and T. macrops in Thailand and a review of the literature. Trans R Soc Trop Med Hyg. 1990;84(6):866-74. doi: 10.1016/0035-9203(90)90111-Q.

15. Mitrakul C, Juzi U, Pongrujikorn W. Antivenom therapy in Russell's viper bite. Am J Clin Pathol. 1991;95(3):412-7. Epub 1991/03/01. doi: 10.1093/ajcp/95.3.412. PubMed PMID: 1996552.

16. Viravan C, Looareesuwan S, Kosakam W, Wuthiekanun V, McCarthy CJ, Stimson AF, et al. A national hospital-based survey of snakes responsible for bites in Thailand. Trans R Soc Trop Med Hyg. 1992;86(1):100-6. doi: 10.1016/0035-9203(92)90463-M.

17. Rojnuckarin P, Mahasandana S, Intragumthornchai T, Sutcharitchan P, Swasdikul D. Prognostic factors of green pit viper bites. Am J Trop Med Hyg. 1998;58(1):22-5. Epub 1998/02/06. doi: 10.4269/ajtmh.1998.58.22. PubMed PMID: 9452286.

18. Rojnuckarin P, Intragumtornchai T, Sattapiboon R, Muanpasitporn C, Pakmanee N, Khow O, et al. The effects of green pit viper (Trimeresurus albolabris and Trimeresurus macrops) venom on the fibrinolytic system in human. Toxicon. 1999;37(5):743-55. Epub 1999/04/29. doi: 10.1016/s0041-0101(98)00214-1. PubMed PMID: 10219986.

19. Wongtongkam N, Wilde H, Sitthi-Amorn C, Ratanabanangkoon K. A study of 225 Malayan pit viper bites in Thailand. Military Medicine. 2005;170(4):342-8. doi: 10.7205/MILMED.170.4.342.

20. Thiansookon A, Rojnuckarin P. Low incidence of early reactions to horse-derived F (ab′) 2 antivenom for snakebites in Thailand. Acta tropica. 2008;105(2):203-5.

21. Chotenimitkhun R, Rojnuckarin P. Systemic antivenom and skin necrosis after green pit viper bites. Clin Toxicol (Phila). 2008;46(2):122-5. Epub 2008/02/09. doi: 10.1080/15563650701266826. PubMed PMID: 18259959.

22. Laohawiriyakamol S, Sangkhathat S, Chiengkriwate P, Patrapinyokul S. Surgery in management of snake envenomation in children. World J Pediatr. 2011;7(4):361-4. Epub 2011/08/31. doi: 10.1007/s12519-011-0282-8. PubMed PMID: 21877258.

23. Tongpoo A, Sriapha C, Pradoo A, Udomsubpayakul U, Srisuma S, Wananukul W, et al. Krait envenomation in Thailand. Therapeutics and Clinical Risk Management. 2018;14:1711-7. doi: 10.2147/TCRM.S169581.

24. Thumtecho S, Tangtrongchitr T, Srisuma S, Kaewrueang T, Rittilert P, Pradoo A, et al. Hematotoxic manifestations and management of green pit viper bites in Thailand. Therapeutics and Clinical Risk Management. 2020;16:695.

25. Malasit P, Warrell DA, Chanthavanich P, Viravan C, Mongkolsapaya J, Singhthong B, et al. Prediction, prevention, and mechanism of early (anaphylactic) antivenom reactions in victims of snake bites. British Medical Journal (Clinical research ed). 1986;292(6512):17-20. doi: 10.1136/bmj.292.6512.17.

26. Pongpit J, Limpawittayakul P, Juntiang J, Akkawat B, Rojnuckarin P. The role of prothrombin time (PT) in evaluating green pit viper (Cryptelytrops sp) bitten patients. Trans R Soc Trop Med Hyg. 2012;106(7):415-8. doi: 10.1016/j.trstmh.2012.04.003.

27. Wongtongkam N, Wilde H, Sitthi-Amorn C, Ratanabanangkoon K. A study of Thai cobra (Naja kaouthia) bites in Thailand. Military medicine. 2005;170(4):336-41.

28. Jiranantakan T, Pantumongkol W, Uansri S, Wisaiprom J, Tantivess S, Leelahavarong P, et al. Budget Impact, Output and Outcome Analysis of Thailand National Antidote Project [Internet]. Health Intervention and Technology Assessment Program, Thailand; 2019 [cited 2020 August 27]. Available from: <https://www.hitap.net/en/research/174746>.

29. Pochanugool C, Limthongkul S, Wilde H. Management of Thai cobra bites with a single bolus of antivenin. Wilderness and Environmental Medicine. 1997;8(1):20-3. doi: 10.1580/1080-6032(1997)008[0020:MOTCBW]2.3.CO;2.

30. Trishnananda M, Oonsombat P, Dumavibhat B, Yongchaiyudha S, Boonyapisit V. Clinical manifestations of cobra bite in the Thai farmer. Am J Trop Med Hyg. 1979;28(1):165-6. doi: 10.4269/ajtmh.1979.28.165.

31. Buranasin P. Snakebites at Maharat Nakhon Ratchasima Regional Hospital. The Southeast Asian journal of tropical medicine and public health. 1993;24(1):186-92.

32. Pochanugool C, Wilde H, Bhanganada K, Chanhome L, Cox MJ, Chaiyabutr N, et al. Venomous snakebite in Thailand II: Clinical experience. Military Medicine. 1998;163(5):318-23. doi: 10.1093/milmed/163.5.318.

33. Riewpaiboon A. Standard cost lists for health economic evaluation in Thailand. Journal of the Medical Association of Thailand= Chotmaihet Thangphaet. 2014;97:S127-34.

34. Adiwinata R, Nelwan EJ. Snakebite in Indonesia. Acta Med Indones. 2015;47(4):358-65. Epub 2016/03/05. PubMed PMID: 26932707.

35. Menteri Kesehatan Republik Indonesia. Peraturan Menteri Kesehatan Republik Indonesia Nomor 63 Tahun 2014 tentang Pengadaan Obat Berdasarkan Katalog Elektronik (E-catalogue). 2014.

36. Watt G, Padre L, Tuazon ML, Hayes CG. Bites by the Philippine cobra (Naja naja philippinensis): an important cause of death among rice farmers. Am J Trop Med Hyg. 1987;37(3):636-9.

37. Edillo FE, Halasa YA, Largo FM, Erasmo JNV, Amoin NB, Alera MTP, et al. Economic cost and burden of dengue in the Philippines. Am J Trop Med Hyg. 2015;92(2):360-6.

38. Department of Health, Republic of the Philippines. Drug Price Reference Index [Internet]. [cited 2021 May 10]. Available from: <https://dpri.doh.gov.ph/index.php?page=search>.

39. Thang VV, Bao TQQ, Tuyen HD, Krumkamp R, Hai LH, Dang NH, et al. Incidence of snakebites in Can Tho Municipality, Mekong Delta, South Vietnam—Evaluation of the responsible snake species and treatment of snakebite envenoming. PLoS Negl Trop Dis. 2020;14(6):e0008430.

40. Flessa S, Dung NT. Costing of services of Vietnamese hospitals: identifying costs in one central, two provincial and two district hospitals using a standard methodology. The international journal of health planning and management. 2004;19(1):63-77.

41. Vongphoumy I, Chanthilat P, Vilayvong P, Blessmann J. Prospective, consecutive case series of 158 snakebite patients treated at Savannakhet provincial hospital, Lao People's Democratic Republic with high incidence of anaphylactic shock to horse derived F (ab') 2 antivenom. Toxicon. 2016;117:13-21.

42. Mahmood MA, Halliday D, Cumming R, Thwin K-T, Zu Kyaw MM, White J, et al. Snakebite incidence in two townships in Mandalay Division, Myanmar. PLoS Negl Trop Dis. 2018;12(7):e0006643.

43. Maternal and Reproductive Health Division, Department of Public Health Myanmar. Costed implementation plan to meet family planning 2020 commitments of Myanmar. Strategic prioritization of implementation 2018-2020 [Internet]. 2018. Available from: <https://www.familyplanning2020.org/sites/default/files/myanmar_cip_2018.10.pdf>.
