# Supplementary material for: Estimating economic and disease burden of snakebite in ASEAN countries using a decision analytic model
Source: PLoS Negl Trop Dis. 2022 Sep 28;16(9):e0010775. doi: 10.1371/journal.pntd.0010775 (PMC9518918; doi:10.1371/journal.pntd.0010775)
Supplement: S2 Appendix — (DOCX) [file pntd.0010775.s002.docx]

**SUPPLEMENTARY MATERIAL**

Estimating economic and disease burden of snakebite in ASEAN countries using a decision analytic model

**S2 Appendix. Estimation of economic and disease burden of post-traumatic stress disorder following snakebite.**

Economic burden of post-traumatic stress disorder (PTSD) following snakebite was estimated as the productivity losses due to PTSD following snakebite. Productivity losses due to PTSD following snakebite were estimated by the number of absent days from work multiplied by daily income. PTSD following snakebite was assumed to last for 41.3 months which was an average duration of chronic PTSD.[1] Lost working days due to PTSD following snakebite was modelled at 36.35 days per year.[2] Thus, lost working days due to PTSD following snakebite were calculated at 125.10 days per case. Productivity losses due to PTSD was valued using a human capital approach by multiplying the time loss due to illness to daily income which was estimated based on the Gross Domestic Product (GDP) per capita of each country.[3]

Disease burden of PTSD following snakebite was estimated as years lived with disability (YLD). YLDs due to PTSD following snakebite envenoming were calculated using the template developed by WHO.[4] YLDs due to PTSD were calculated from the duration of PTSD of 41.3 months multiplied by disability weight of 0.523 for severe anxiety.[1, 5]

**References**

1. Kessler RC, Aguilar-Gaxiola S, Alonso J, Benjet C, Bromet EJ, Cardoso G, et al. Trauma and PTSD in the WHO world mental health surveys. European journal of psychotraumatology. 2017;8(sup5):1353383.

2. Ferry FR, Brady SE, Bunting BP, Murphy SD, Bolton D, O'Neill SM. The economic burden of PTSD in Northern Ireland. Journal of traumatic stress. 2015;28(3):191-7.

3. World Bank. GDP per capita (current LCU) [Internet]. 2019. Available from: <https://data.worldbank.org/indicator/NY.GDP.PCAP.CN>.

4. Mathers CD, Vos T, Lopez AD, Salomon J, Ezzati M. National burden of disease studies: a practical guide. Geneva: World Health Organization. 2001.

5. Salomon JA, Haagsma JA, Davis A, de Noordhout CM, Polinder S, Havelaar AH, et al. Disability weights for the Global Burden of Disease 2013 study. The Lancet Global Health. 2015;3(11):e712-e23.
