# Supplementary material for: Estimating economic and disease burden of snakebite in ASEAN countries using a decision analytic model
Source: PLoS Negl Trop Dis. 2022 Sep 28;16(9):e0010775. doi: 10.1371/journal.pntd.0010775 (PMC9518918; doi:10.1371/journal.pntd.0010775)
Supplement: S1 Table. Input parameters for estimating economic and disease burden of snakebite in ASEAN countries — (DOCX) [file pntd.0010775.s003.docx]

**SUPPLEMENTARY MATERIAL**

Estimating economic and disease burden of snakebite in ASEAN countries using a decision analytic model

**S1 Table. Input parameters for estimating economic and disease burden of snakebite in ASEAN countries.**

| **Parameters (Distribution)** | **Malaysia** | **Thailand** | **Indonesia** | **Philippines** | **Vietnam** | **Lao PDR** | **Myanmar** |
| --- | --- | --- | --- | --- | --- | --- | --- |
| **Epidemiological parameters** |  |  |  |  |  |  |  |
| Total population, million people[1] | 32 | 70 | 271 | 108 | 96 | 7 | 54 |
| Incidence of snakebite per 100,000 population (Beta)[2-6] | 10.7  (10.3 to 11.1) | 12.52  (12.25 to 12.78) | 49.9  (49.6 to 50.2) | 12.4  (10.5 to 14.5) | 48.5  (19.5 to 99.8) | 200.0  (196.7 to 203.3) | 39.0  (38.4 to 39.5) |
| Probability of snakebite victims seeking conventional treatment only (Beta)[5, 7] | 0.94  (0.93 to 0.95) | 0.965  (0.961 to 0.969) | 0.75 | 0.00 | 0.00 | 0.00 | 0.34  (0.32 to 0.35) |
| Probability of snakebite victims firstly seeking traditional treatment then switching to conventional treatment (Beta)[5, 6, 8-10] | 0.06  (0.03 to 0.10) | 0.03  (0.02 to 0.06) | 0.00 | 0.27 | 0.43  (0.29 to 0.59) | 0.10 | 0.07  (0.05 to 0.08) |
| Probability of systemic envenoming indicated for antivenom treatment for victims seeking conventional treatment (Beta)[5, 6, 8, 10-25] | 0.14  (0.08 to 0.23) | 0.59  (0.42 to 0.74) | 0.40  (0.26 to 0.57) | 0.16  (0.12 to 0.21) | 0.90  (0.87 to 0.93) | 0.27  (0.20 to 0.35) | 0.79  (0.76 to 0.81) |
| Probability of systemic envenoming for victims seeking traditional treatment | 0.08 | 0.42 | 0.26 | 0.12 | 0.87 | 0.20 | 0.76 |
| Probability of antivenom given to victims with systemic envenoming seeking care at the healthcare facilities* | 1.00 | 1.00 | 0.12 | 0.77 | 0.83 | 0.33 | 1.00 |
| Probability of adverse reaction following antivenom treatment (Beta)[5, 17, 25, 26] | 0.10 | 0.04  (0.02 to 0.07) | 0.40 | 0.05 | 0.22  (0.03 to 0.60) | 0.53  (0.38 to 0.69) | 0.08  (0.06 to 0.10) |
| Probability of death in snakebite victims without systemic envenoming[4-6, 11, 17, 24, 25] | 0.00 | 0.00 | 0.00 | 0.00 | 0.00 | 0.00 | 0.00 |
| Probability of death in systemic envenoming treated with antivenom (Beta)[2, 5, 24, 25, 27, 28] | 0.002  (0.001 to 0.013) | 0.0008  (0.0005 to 0.0012) | 0.09  (0.07 to 0.12) | 0.07  (0.01 to 0.18) | 0.015  (0.008 to 0.025) | 0.05  (0.01 to 0.16) | 0.068  (0.061 to 0.074) |
| Relative risk of death when antivenoms are not available (Log-normal)[27] | 2.33  (1.26 to 4.06) | 2.33  (1.26 to 4.06) | 2.33  (1.26 to 4.06) | 2.33  (1.26 to 4.06) | 2.33  (1.26 to 4.06) | 2.33  (1.26 to 4.06) | 2.33  (1.26 to 4.06) |
| Probability of death in systemic envenoming treated in hospital without antivenom (Beta) | 0.005 | 0.0018 | 0.21 | 0.16 | 0.035 | 0.11 | 0.16 |
| Probability of death in systemic envenoming not treated in hospital (Beta) | 0.030 | 0.0028 | 0.28 | 0.43 | 0.057 | 0.37 | 0.17 |
| Probability of digit amputation due to snakebite envenoming (Beta)[3, 5, 6, 8, 9, 14, 17, 19, 22, 25, 29-32] | 0.00 | 0.003  (0.001 to 0.012) | 0.01  (0.005 to 0.02) | 0.007 | 0.00 | 0.05  (0.01 to 0.16) | 0.00 |
| Probability of limb amputation due to snakebite envenoming (Beta)[3, 5, 6, 8, 9, 14, 17, 19, 22, 25, 29-32] | 0.00 | 0.00 | 0.01  (0.005 to 0.02) | 0.003 | 0.00 | 0.02  (0.001 to 0.12) | 0.00 |
| **Disability weight** |  |  |  |  |  |  |  |
| Disability weight for victims not indicated for antivenom treatment (Beta)[33] | 0.006  (0.002 to 0.012) | 0.006  (0.002 to 0.012) | 0.006  (0.002 to 0.012) | 0.006  (0.002 to 0.012) | 0.006  (0.002 to 0.012) | 0.006  (0.002 to 0.012) | 0.006  (0.002 to 0.012) |
| Disability weight for victims indicated for antivenom treatment (Beta)[33] | 0.163  (0.109 to 0.227) | 0.163  (0.109 to 0.227) | 0.163  (0.109 to 0.227) | 0.163  (0.109 to 0.227) | 0.163  (0.109 to 0.227) | 0.163  (0.109 to 0.227) | 0.163  (0.109 to 0.227) |
| Disability weight for digit amputation (Beta)[33] | 0.005  (0.002 to 0.010) | 0.005  (0.002 to 0.010) | 0.005  (0.002 to 0.010) | 0.005  (0.002 to 0.010) | 0.005  (0.002 to 0.010) | 0.005  (0.002 to 0.010) | 0.005  (0.002 to 0.010) |
| Disability weight for limb amputation (Beta)[33] | 0.039  (0.024 to 0.059) | 0.039  (0.024 to 0.059) | 0.039  (0.024 to 0.059) | 0.039  (0.024 to 0.059) | 0.039  (0.024 to 0.059) | 0.039  (0.024 to 0.059) | 0.039  (0.024 to 0.059) |
| **Duration of disease** |  |  |  |  |  |  |  |
| Length of stay for victims not indicated for antivenom treatment, day | 1 | 1 | 1 | 1 | 1 | 1 | 1 |
| Length of stay for victims indicated for antivenom treatment (Gamma)[9-11, 13, 20, 21, 29, 31] | 6.1 (4.1 to 8.0) | 3.5 (2.6 to 4.4) | 6.1 (4.1 to 8.0) | 6.1 (4.1 to 8.0) | 6.1 (4.1 to 8.0) | 6.1 (4.1 to 8.0) | 6.1 (4.1 to 8.0) |
| **Unit costs, USD** |  |  |  |  |  |  |  |
| Unit cost of hospitalization for victims not indicated for antivenom treatment (Gamma)[34-39] | 150  (135 to 164) | 103  (93 to 114) | 175  (158 to 193) | 68  (61 to 75) | 31  (28 to 34) | 69  (62 to 76) | 36  (32 to 39) |
| Unit cost of hospitalization for victims not indicated for antivenom treatment (Gamma)[34-39] | 751  (676 to 826) | 332  (290 to 354) | 1,007  (907 to 1,108) | 408  (367 to 449) | 173  (155 to 190) | 372  (335 to 410) | 210  (189 to 231) |
| Unit cost of antivenom treatment (Gamma)[34, 35, 37, 40] | 992  (893 to 1,091) | 217  (196 to 239) | 773  (695 to 850) | 312  (281 to 343) | 68  (61 to 75) | 202  (181 to 222) | 326  (293 to 359) |
| Unit cost of antivenom logistics costs, percentage of antivenom price[40] | 0.05 | 0.05 | 0.05 | 0.05 | 0.05 | 0.05 | 0.05 |
| Unit cost of adverse reaction management (Gamma)[34, 35, 37, 41] | 3.9 (3.5 to 4.3) | 3.4 (3.1 to 3.7) | 3.8 (3.5 to 4.2) | 4.4 (4.0 to 4.9) | 5.1 (4.6 to 5.7) | 5.1 (4.6 to 5.7) | 0.76 (0.69 to 0.84) |
| Unit cost of digit amputation (Gamma)[40] | 96 (86 to 106) | 96 (86 to 106) | 96 (86 to 106) | 96 (86 to 106) | 96 (86 to 106) | 52 (47 to 57) | 96 (86 to 106) |
| Unit cost of limb amputation (Gamma)[40] | 154 (138 to 169) | 154 (138 to 169) | 154 (138 to 169) | 154 (138 to 169) | 154 (138 to 169) | 158 (143 to 174) | 154 (138 to 169) |
| Unit cost of traditional healer | 0 | 0 | 0 | 0 | 0 | 0 | 0 |
| Unit cost of transportation (Gamma)[34, 42, 43] | 5.9 (5.3 to 6.5) | 3.4 (3.1 to 3.6) | 7.8 (7.0 to 8.6) | 5.8 (5.2 to 6.4) | 21 (19 to 23) | 4.7 (4.2 to 5.1) | 20 (18 to 22) |
| Unit cost of additional food (Gamma)[34, 42, 44] | 2.7 (2.4 to 2.9) | 1.2 (1.0 to 1.3) | 2.3 (2.1 to 2.6) | 2.3 (2.1 to 2.6) | 6.5 (5.9 to 7.2) | 2.4 (2.3 to 2.5) | 3.3 (3.0 to 3.6) |
| **Others** |  |  |  |  |  |  |  |
| Discount rate[45-47] | 0.03  (0.00 to 0.05) | 0.03  (0.00 to 0.06) | 0.03  (0.00 to 0.06) | 0.03  (0.00 to 0.06) | 0.03  (0.00 to 0.06) | 0.03  (0.00 to 0.06) | 0.03  (0.00 to 0.06) |
| GDP per capita, USD[48] | 11,415 | 7,808 | 4,136 | 3,485 | 2,715 | 2,625 | 1,421 |
| GDP per capita annual growth, %[49] | 0.03 | 0.02 | 0.04 | 0.05 | 0.06 | 0.03 | 0.02 |
| Number of relatives or family members who companied snakebite victims | 1 | 1 | 1 | 2 | 1 | 1 | 2 |

Parameters are presented as base-case value (range). Costs are presented as USD where 1 USD equals to 4.14 Malaysian Ringgits, 31.05 Thai Bahts, 14,147.67 Indonesian Rupees, 51.80 Philippine Pesos, 23,050.24 Vietnamese Dongs, 8,679.41 Lao Kips, and 1,518.26 Myanmar Kyats. *– Probability of antivenom given to victims with systemic envenoming seeking care at the healthcare facilities was determined by the number of antivenom treatments available divided by the number of snakebite victims needed antivenom treatment who sought care at the healthcare facilities.

**References**

1. World Bank. Population, total [Internet]. 2019. Available from: <https://data.worldbank.org/indicator/SP.POP.TOTL>.

2. Sivaganabalan R, Ismail AK, Salleh MS, Mohan K, Tan CH, Adnan A. Guideline on the Management of Snakebites. Ministry of Health Malaysia; 2017.

3. National Health Security Office, Thailand. Report on the creation of the National Health Security for fiscal year BE 2562 (AD 2019). Bangkok, Thailand; 2019.

4. Watt G, Padre L, Tuazon ML, Hayes CG. Bites by the Philippine cobra (Naja naja philippinensis): an important cause of death among rice farmers. Am J Trop Med Hyg. 1987;37(3):636-9.

5. Mahmood MA, Halliday D, Cumming R, Thwin K-T, Zu Kyaw MM, White J, et al. Snakebite incidence in two townships in Mandalay Division, Myanmar. PLoS Negl Trop Dis. 2018;12(7):e0006643.

6. Thang VV, Bao TQQ, Tuyen HD, Krumkamp R, Hai LH, Dang NH, et al. Incidence of snakebites in Can Tho Municipality, Mekong Delta, South Vietnam—Evaluation of the responsible snake species and treatment of snakebite envenoming. PLoS Negl Trop Dis. 2020;14(6):e0008430.

7. Longbottom J, Shearer FM, Devine M, Alcoba G, Chappuis F, Weiss DJ, et al. Vulnerability to snakebite envenoming: A global mapping of hotspots. The Lancet. 2018;392(10148):673-84.

8. Mitrakul C, Dhamkrong-At A, Futrakul P, Thisyakorn C, Vongsrisart K, Varavithya C, et al. Clinical features of neurotoxic snake bite and response to antivenom in 47 children. Am J Trop Med Hyg. 1984;33(6):1258-66. Epub 1984/11/01. doi: 10.4269/ajtmh.1984.33.1258. PubMed PMID: 6507733.

9. Wongtongkam N, Wilde H, Sitthi-Amorn C, Ratanabanangkoon K. A study of Thai cobra (Naja kaouthia) bites in Thailand. Military medicine. 2005;170(4):336-41.

10. Wongtongkam N, Wilde H, Sitthi-Amorn C, Ratanabanangkoon K. A study of 225 Malayan pit viper bites in Thailand. Military Medicine. 2005;170(4):342-8. doi: 10.7205/MILMED.170.4.342.

11. Shafie NA, Fauzi H, Wahab M, Senek M, Ismail A. The prevalence of hypersensitivity reactions to snake antivenoms administered in sultanah nur zahirah hospital from 2013 to 2016. Med J Malaysia. 2020;75(3):217.

12. Hutton RA, Looareesuwanzj S, Ho M, Silamut K, Chanthavanich P, Karbwang J, et al. Arboreal green pit vipers (genus Trimeresurus) of south-east Asia: Bites by T. albolabris and T. macrops in Thailand and a review of the literature. Trans R Soc Trop Med Hyg. 1990;84(6):866-74. doi: 10.1016/0035-9203(90)90111-Q.

13. Mitrakul C, Juzi U, Pongrujikorn W. Antivenom therapy in Russell's viper bite. Am J Clin Pathol. 1991;95(3):412-7. Epub 1991/03/01. doi: 10.1093/ajcp/95.3.412. PubMed PMID: 1996552.

14. Viravan C, Looareesuwan S, Kosakam W, Wuthiekanun V, McCarthy CJ, Stimson AF, et al. A national hospital-based survey of snakes responsible for bites in Thailand. Trans R Soc Trop Med Hyg. 1992;86(1):100-6. doi: 10.1016/0035-9203(92)90463-M.

15. Rojnuckarin P, Mahasandana S, Intragumthornchai T, Sutcharitchan P, Swasdikul D. Prognostic factors of green pit viper bites. Am J Trop Med Hyg. 1998;58(1):22-5. Epub 1998/02/06. doi: 10.4269/ajtmh.1998.58.22. PubMed PMID: 9452286.

16. Rojnuckarin P, Intragumtornchai T, Sattapiboon R, Muanpasitporn C, Pakmanee N, Khow O, et al. The effects of green pit viper (Trimeresurus albolabris and Trimeresurus macrops) venom on the fibrinolytic system in human. Toxicon. 1999;37(5):743-55. Epub 1999/04/29. doi: 10.1016/s0041-0101(98)00214-1. PubMed PMID: 10219986.

17. Thiansookon A, Rojnuckarin P. Low incidence of early reactions to horse-derived F (ab′) 2 antivenom for snakebites in Thailand. Acta tropica. 2008;105(2):203-5.

18. Chotenimitkhun R, Rojnuckarin P. Systemic antivenom and skin necrosis after green pit viper bites. Clin Toxicol (Phila). 2008;46(2):122-5. Epub 2008/02/09. doi: 10.1080/15563650701266826. PubMed PMID: 18259959.

19. Laohawiriyakamol S, Sangkhathat S, Chiengkriwate P, Patrapinyokul S. Surgery in management of snake envenomation in children. World J Pediatr. 2011;7(4):361-4. Epub 2011/08/31. doi: 10.1007/s12519-011-0282-8. PubMed PMID: 21877258.

20. Tongpoo A, Sriapha C, Pradoo A, Udomsubpayakul U, Srisuma S, Wananukul W, et al. Krait envenomation in Thailand. Therapeutics and Clinical Risk Management. 2018;14:1711-7. doi: 10.2147/TCRM.S169581.

21. Thumtecho S, Tangtrongchitr T, Srisuma S, Kaewrueang T, Rittilert P, Pradoo A, et al. Hematotoxic manifestations and management of green pit viper bites in Thailand. Therapeutics and Clinical Risk Management. 2020;16:695.

22. Malasit P, Warrell DA, Chanthavanich P, Viravan C, Mongkolsapaya J, Singhthong B, et al. Prediction, prevention, and mechanism of early (anaphylactic) antivenom reactions in victims of snake bites. British Medical Journal (Clinical research ed). 1986;292(6512):17-20. doi: 10.1136/bmj.292.6512.17.

23. Pongpit J, Limpawittayakul P, Juntiang J, Akkawat B, Rojnuckarin P. The role of prothrombin time (PT) in evaluating green pit viper (Cryptelytrops sp) bitten patients. Trans R Soc Trop Med Hyg. 2012;106(7):415-8. doi: 10.1016/j.trstmh.2012.04.003.

24. Adiwinata R, Nelwan EJ. Snakebite in Indonesia. Acta Med Indones. 2015;47(4):358-65. Epub 2016/03/05. PubMed PMID: 26932707.

25. Vongphoumy I, Chanthilat P, Vilayvong P, Blessmann J. Prospective, consecutive case series of 158 snakebite patients treated at Savannakhet provincial hospital, Lao People's Democratic Republic with high incidence of anaphylactic shock to horse derived F (ab') 2 antivenom. Toxicon. 2016;117:13-21.

26. Le Khac Q. Clinical evaluation of snakebites in Vietnam: A study from Cho Ray hospital. 2004.

27. Habib AG, Warrell DA. Antivenom therapy of carpet viper (Echis ocellatus) envenoming: effectiveness and strategies for delivery in West Africa. Toxicon. 2013;69:82-9.

28. Jiranantakan T, Pantumongkol W, Uansri S, Wisaiprom J, Tantivess S, Leelahavarong P, et al. Budget Impact, Output and Outcome Analysis of Thailand National Antidote Project [Internet]. Health Intervention and Technology Assessment Program, Thailand; 2019 [cited 2020 August 27]. Available from: <https://www.hitap.net/en/research/174746>.

29. Pochanugool C, Limthongkul S, Wilde H. Management of Thai cobra bites with a single bolus of antivenin. Wilderness and Environmental Medicine. 1997;8(1):20-3. doi: 10.1580/1080-6032(1997)008[0020:MOTCBW]2.3.CO;2.

30. Trishnananda M, Oonsombat P, Dumavibhat B, Yongchaiyudha S, Boonyapisit V. Clinical manifestations of cobra bite in the Thai farmer. Am J Trop Med Hyg. 1979;28(1):165-6. doi: 10.4269/ajtmh.1979.28.165.

31. Buranasin P. Snakebites at Maharat Nakhon Ratchasima Regional Hospital. The Southeast Asian journal of tropical medicine and public health. 1993;24(1):186-92.

32. Pochanugool C, Wilde H, Bhanganada K, Chanhome L, Cox MJ, Chaiyabutr N, et al. Venomous snakebite in Thailand II: Clinical experience. Military Medicine. 1998;163(5):318-23. doi: 10.1093/milmed/163.5.318.

33. Salomon JA, Haagsma JA, Davis A, de Noordhout CM, Polinder S, Havelaar AH, et al. Disability weights for the Global Burden of Disease 2013 study. The Lancet Global Health. 2015;3(11):e712-e23.

34. Attorney General’s Chambers Malaysia. Fees (Medical) (Cost of Services) Order 2014. Federal Government Gazette; 2014.

35. Menteri Kesehatan Republik Indonesia. Peraturan Menteri Kesehatan Republik Indonesia Nomor 63 Tahun 2014 tentang Pengadaan Obat Berdasarkan Katalog Elektronik (E-catalogue). 2014.

36. Edillo FE, Halasa YA, Largo FM, Erasmo JNV, Amoin NB, Alera MTP, et al. Economic cost and burden of dengue in the Philippines. Am J Trop Med Hyg. 2015;92(2):360-6.

37. Department of Health, Republic of the Philippines. Drug Price Reference Index [Internet]. [cited 2021 May 10]. Available from: <https://dpri.doh.gov.ph/index.php?page=search>.

38. Maternal and Reproductive Health Division, Department of Public Health Myanmar. Costed implementation plan to meet family planning 2020 commitments of Myanmar. Strategic prioritization of implementation 2018-2020 [Internet]. 2018. Available from: <https://www.familyplanning2020.org/sites/default/files/myanmar_cip_2018.10.pdf>.

39. Flessa S, Dung NT. Costing of services of Vietnamese hospitals: identifying costs in one central, two provincial and two district hospitals using a standard methodology. The international journal of health planning and management. 2004;19(1):63-77.

40. Riewpaiboon A. Standard cost lists for health economic evaluation in Thailand. Journal of the Medical Association of Thailand= Chotmaihet Thangphaet. 2014;97:S127-34.

41. Pharmaceutical Services Programme, Ministry of Health Malaysia,. Consumer Price Guide [Internet]. Available from: <https://www.pharmacy.gov.my/v2/en/apps/drug-price>.

42. Chong HY, Lim YH, Prawjaeng J, Tassaneeyakul W, Mohamed Z, Chaiyakunapruk N. Cost-effectiveness analysis of HLA-B* 58: 01 genetic testing before initiation of allopurinol therapy to prevent allopurinol-induced Stevens–Johnson syndrome/toxic epidermal necrolysis in a Malaysian population. Pharmacogenetics and genomics. 2018;28(2):56-67.

43. Riewpaiboon A. Economic burden of hand, foot, and mouth disease in Vietnam; An evidence for priority setting and efficiency management.

44. Vodicka E, Zimmermann M, Lopez AL, Silva MW, Gorgolon L, Kohei T, et al. Japanese encephalitis vaccination in the Philippines: A cost-effectiveness analysis comparing alternative delivery strategies. Vaccine. 2020;38(13):2833-40.

45. Division of Pharmaceutical Sciences, Ministry of Health Malaysia (MOH). Pharmacoeconomic guideline for Malaysia. Putrajaya, Malaysia: 2012.

46. Teerawattananon Y. Guidelines for health technology assessment in Thailand (second edition). J Med Assoc Thai. 2014;97(5):S4–9.

47. Indonesian Health Technology Assessment Committee MoHotRoI. Health Technology Assessment (HTA) guideline.

48. World Bank. GDP per capita (current LCU) [Internet]. 2019. Available from: <https://data.worldbank.org/indicator/NY.GDP.PCAP.CN>.

49. World Bank. GDP per capita growth (annual %) [Internet]. 2019. Available from: <https://data.worldbank.org/indicator/NY.GDP.PCAP.KD.ZG>.
