# Supplementary material for: Estimating economic and disease burden of snakebite in ASEAN countries using a decision analytic model
Source: PLoS Negl Trop Dis. 2022 Sep 28;16(9):e0010775. doi: 10.1371/journal.pntd.0010775 (PMC9518918; doi:10.1371/journal.pntd.0010775)
Supplement: S2 Table — (DOCX) [file pntd.0010775.s004.docx]

**SUPPLEMENTARY MATERIAL**

Estimating economic and disease burden of snakebite in ASEAN countries using a decision analytic model

**S2 Table. Estimated annual epidemiological and disease burden of snakebite in ASEAN countries.**

|  | Incidence per 100,000 | Mortality per 100,000 | YLLs | YLDs for snakebite episode | YLDs for amputation |
| --- | --- | --- | --- | --- | --- |
| Malaysia | 10.68 (10.34-11.06) | 0.006 (0.001-0.019) | 50 (0-151) | 1 (1-3) | 0 |
| Thailand | 12.52 (12.24-12.79) | 0.006 (0.003-0.009) | 102 (51-178) | 8 (4-13) | 0.21 (0.01-0.91) |
| Indonesia | 49.88 (49.62-50.14) | 3.90 (1.85-8.34) | 262,302 (124,650-561,145) | 149 (77-252) | 437 (170-868) |
| Philippines | 12.37 (10.59-14.59) | 0.51 (0.25-1.02) | 13,311 (6,624-26,641) | 5 (3-8) | 2 (1-3) |
| Vietnam | 48.46 (18.14-94.35) | 1.72 (0.51-4.60) | 40,136 (11,869-107,679) | 114 (38-258) | 0 |
| Lao PDR | 200.00 (196.82-203.23) | 14.04 (7.12-28.03) | 24,468 (12,420-48,837) | 8 (5-13) | 56 (5-176) |
| Myanmar | 38.97 (38.16-39.86) | 3.97 (2.41-7.08) | 50,786 (30,877-90,632) | 44 (27-67) | 0 |
| Total | 38.03 (32.89-45.62) | 2.49 (1.19-5.32) | 391,154 (186,491-835,263) | 330 (154-613) | 495 (175-1,049) |

Estimates are presented as base-case estimates with their 95% credibility interval (in parentheses) based on probabilistic sensitivity analysis. Abbreviations: DALYs – disability-adjusted life years; YLDs – years lived with disabilities; YLLs – years of life lost.
