# Supplementary material for: Estimating economic and disease burden of snakebite in ASEAN countries using a decision analytic model
Source: PLoS Negl Trop Dis. 2022 Sep 28;16(9):e0010775. doi: 10.1371/journal.pntd.0010775 (PMC9518918; doi:10.1371/journal.pntd.0010775)
Supplement: S3 Table — (DOCX) [file pntd.0010775.s005.docx]

**SUPPLEMENTARY MATERIAL**

Estimating economic and disease burden of snakebite in ASEAN countries using a decision analytic model

**S3 Table. Estimated annual epidemiological and disease burden of snakebite envenoming per case in ASEAN countries.**

|  | Mortality rate | Amputation rate | DALYs per case | Direct costs per case, USD | Indirect costs per case, USD | Total costs per case, USD |
| --- | --- | --- | --- | --- | --- | --- |
| Malaysia | 0.004 (0.001-0.013) | - | 0.11 (0.00-0.32) | 1,736 (1,609-1,874) | 1,649 (377-4,360) | 3,386 (2,110-6,120) |
| Thailand | 0.001 (0.000-0.001) | 0.0003 (0.000-0.001) | 0.02 (0.01-0.03) | 564 (492-641) | 297 (223-377) | 861 (739-998) |
| Indonesia | 0.213 (0.107-0.420) | 0.016 (0.008-0.028) | 5.30 (2.68-10.45) | 929 (708-1,157) | 38,867 (19,690-76,612) | 39,796 (20,616-77,511) |
| Philippines | 0.313 (0.158-0.642) | 0.007 (0.004-0.008) | 7.59 (3.83-15.52 | 240 (193-292) | 46,833 (23,709-95,880) | 47,072 (23,924-96,113) |
| Vietnam | 0.040 (0.021-0.077) | - | 0.98 (0.51-1.86) | 156 (103-216) | 6,337 (3,337-12,025) | 6,493 (3,534-12,166) |
| Lao PDR | 0.332 (0.167-0.668) | 0.047 (0.007-0.115) | 8.10 (4.10-16.23) | 32 (25-40) | 26,508 (13,351-53,172) | 26,540 (13,390-53,199) |
| Myanmar | 0.132 (0.080-0.235) | - | 3.12 (1.90-5.56) | 260 (231-300) | 4,591 (2,819-8,125) | 4,851 (3,072-8,378) |
| ASEAN | 0.135 (0.089-0.234) | 0.008 (0.004-0.014) | 3.33 (2.19-5.78) | 519 (381-697) | 20,667 (12,391-38,973) | 21,186 (12,905-39,710) |

Estimates are presented as base-case estimates with their 95% credibility interval (in parentheses) based on probabilistic sensitivity analysis. Costs are presented as 2019 USD where 1 USD = 14,147.67 Indonesian Rupees = 51.80 Philippine Pesos = 23,050.24 Vietnamese Dong = 8,679.41 Lao Kip = 1,518.26 Myanmar Kyat. Abbreviations: DALYs – disability-adjusted life years; USD – US Dollar.
