# Supplementary material for: Estimating economic and disease burden of snakebite in ASEAN countries using a decision analytic model
Source: PLoS Negl Trop Dis. 2022 Sep 28;16(9):e0010775. doi: 10.1371/journal.pntd.0010775 (PMC9518918; doi:10.1371/journal.pntd.0010775)
Supplement: S4 Table — (DOCX) [file pntd.0010775.s006.docx]

**SUPPLEMENTARY MATERIAL**

Estimating economic and disease burden of snakebite in ASEAN countries using a decision analytic model

**S4 Table. Estimated annual economic and disease burden of post-traumatic stress disorder following snakebite.**

|  | PTSD, n | YLDs | Productivity losses, x1000 USD |
| --- | --- | --- | --- |
| Malaysia | 98 (69-155) | 168 (81-324) | 382 (206-678) |
| Thailand | 464 (161-977) | 794 (239-1,797) | 1,228 (371-2,796) |
| Indonesia | 4,738 (2,480-8,514) | 8,103 (3,144-16,702) | 6,783 (2,866-13,287) |
| Philippines | 334 (250-481) | 426 (238-662) | 407 (229-667) |
| Vietnam | 3,103 (554-8,792) | 5,306 (867-16,305) | 2,994 (499-8,962) |
| Lao PDR | 390 (257-587) | 666 (304-1,174) | 351 (181-618) |
| Myanmar | 1,167 (880-1,448) | 1,995 (997-3,070) | 563 (319-856) |
| Total | 10,293 (4,651-20,954) | 17,458 (5,869-40,035) | 12,708 (4,670-27,864) |

Estimates are presented as base-case estimates as x1000 USD with their 95% credibility interval (in parentheses) based on probabilistic sensitivity analysis. Costs are presented as 2019 USD where 1 USD = 14,147.67 Indonesian Rupees = 51.80 Philippine Pesos = 23,050.24 Vietnamese Dong = 8,679.41 Lao Kip = 1,518.26 Myanmar Kyat. Abbreviations: PTSD – post-traumatic stress disorder; USD – US Dollar, YLDs – years lived with disabilities.
