# Supplementary material for: Estimating economic and disease burden of snakebite in ASEAN countries using a decision analytic model
Source: PLoS Negl Trop Dis. 2022 Sep 28;16(9):e0010775. doi: 10.1371/journal.pntd.0010775 (PMC9518918; doi:10.1371/journal.pntd.0010775)
Supplement: S1 Fig — (DOCX) [file pntd.0010775.s007.docx]

**SUPPLEMENTARY MATERIAL**

Estimating economic and disease burden of snakebite in ASEAN countries using a decision analytic model

**S1 Fig. One-way sensitivity analysis of economic burden.**
