# Supplementary material for: Estimating economic and disease burden of snakebite in ASEAN countries using a decision analytic model
Source: PLoS Negl Trop Dis. 2022 Sep 28;16(9):e0010775. doi: 10.1371/journal.pntd.0010775 (PMC9518918; doi:10.1371/journal.pntd.0010775)
Supplement: S3 Fig — Estimated disease burden of snakebite from this study (shown in purple) was compared to the disease burden of neglected tropical diseases in seven ASEAN countries that were estimated in the Global Burden of Disease 2019 study. (DOCX) [file pntd.0010775.s009.docx]

**SUPPLEMENTARY MATERIAL**

Estimating economic and disease burden of snakebite in ASEAN countries using a decision analytic model

**S3 Fig. Comparison of annual disease burden of neglected tropical diseases in ASEAN.**

Estimated disease burden of snakebite from this study (shown in purple) was compared to the disease burden of neglected tropical diseases in seven ASEAN countries that were estimated in the Global Burden of Disease 2019 study.[1]

**References**

1. Vos T, Lim SS, Abbafati C, Abbas KM, Abbasi M, Abbasifard M, et al. Global burden of 369 diseases and injuries in 204 countries and territories, 1990–2019: a systematic analysis for the Global Burden of Disease Study 2019. The Lancet. 2020;396(10258):1204-22.
