# Supplementary material for: Estimating economic and disease burden of snakebite in ASEAN countries using a decision analytic model
Source: PLoS Negl Trop Dis. 2022 Sep 28;16(9):e0010775. doi: 10.1371/journal.pntd.0010775 (PMC9518918; doi:10.1371/journal.pntd.0010775)
Supplement: Attachment — Submitted filename: 1. Author response letter_burden of snakebite in ASEAN.docx [file pntd.0010775.s010.docx]

**AUTHOR RESPONSE LETTER**

**PNTD-D-22-00588: Estimating economic and disease burden of snakebite in ASEAN countries using a decision analytic model**

Dear Editor,

On behalf of all the authors, I would like to resubmit our manuscript entitled: “Estimating economic and disease burden of snakebite in ASEAN countries using a decision analytic model” (PNTD-D-22-00588). The comments forwarded from the editor and reviewers are highly appreciated and contain useful suggestions. We have revised our manuscript and responded point-by-point to each issue raised by the reviewers. We do appreciate all valuable comments from the reviewers and have tried our best to incorporate them in this revised version.

All authors have contributions that meets criteria for authorship and all authors have signed a statement attesting authorship, disclosing all potential conflicts of interest, and releasing the copyright should the manuscript be accepted for publication. We attest that this work is original and has not been published and is not being considered for publication elsewhere, in any language.

Thank you for considering this paper for publication.

Sincerely yours,

Nathorn Chaiyakunapruk, PhD

Department of Pharmacotherapy, College of Pharmacy, University of Utah, 30 2000 E, Salt Lake City, Utah, 84112

Email: [Nathorn.Chaiyakunapruk@utah.edu](mailto:Nathorn.Chaiyakunapruk@utah.edu)

Tel: +1.801.585.3092

Suthira Taychakhoonavudh, PhD

Department of Social and Administrative Pharmacy, Faculty of Pharmaceutical Sciences, Chulalongkorn University, 254 Phayathai Road Pathumwan, Bangkok, 10330, Thailand

Email: [Suthira.T@chula.ac.th](mailto:Suthira.T@chula.ac.th)

Tel: +662-218-8386-90

**REVIEWER’S COMMENTS**

**METHODS**

**Reviewer #1: I am not a Health Economist and my review is therefore limited in its scope. I sincerely hope that the Editor has sought a review from a suitable qualified Health Economist**

**-The objectives of the study were clearly articulated and a clear testable hypothesis was stated.**

Response: Thank you for your valuable comments. We have revised the manuscript to address your suggestions and provided point-by-point responses below.

**-The study design is appropriate to address the stated objectives - but does necessarily (due to lack of primary data) make many assumptions. These assumptions include data on:**

**- incidence of PTSD following snake envenoming - the authors need to add a reference for this because I don't know any reliable study on this topic**

Response: We have added a reference for the incidence of PTSD following snakebite envenoming (Khosrojerdi H, Amini M. Acute and delayed stress symptoms following snakebite. Asia Pacific Journal of Medical Toxicology. 2013;2(4):140-4.).

**- days lost post-envenoming - how was this calculated? This needs to be added to the supplementary data**

Response: We have explained how we calculated days lost post-envenoming due to PTSD in S2 appendix in the supplementary data. We have explained that;

*Economic burden of post-traumatic stress disorder (PTSD) following snakebite was estimated as the productivity losses due to PTSD following snakebite. Productivity losses due to PTSD following snakebite were estimated by the number of absent days from work multiplied by daily income. PTSD following snakebite was assumed to last for 41.3 months which was an average duration of chronic PTSD.[44] Lost working days due to PTSD following snakebite was modelled at 36.35 days per year.[45] Thus, lost working days due to PTSD following snakebite were calculated at 125.10 days per case. Productivity losses due to PTSD was valued using a human capital approach by multiplying the time loss due to illness to daily income which was estimated based on the Gross Domestic Product (GDP) per capita of each country.[46]*

**-The populations examined in this study are clearly described and appropriate.**

**-The sample size is sufficient to ensure adequate power.**

**-Appropriate statistical analysis was used to support conclusions.**

**-Based upon the manuscript I believe that there no concerns about ethical or regulatory requirements**

Response: Thank you for your time reviewing our manuscript.

**Reviewer #2: The objective is clearly stated. The study design is appropriate.**

**Only one-year incidence of snakebite was used for the calculation. When available, average incidence from 3-5 years should be used.**

Response: We consulted with experts in snakebite in ASEAN countries and made a decision to apply the most recent incidence of snakebite in the analysis to reflect the current situation in the region.

**Reviewer #3: Estimating economic and disease burden of snakebite in ASEAN countries using a**

**decision analytic model**

**It has been very stimulating and enlightening to review this very interesting and timely paper on economic and disease burden of snakebite in ASEAN countries. The study has clear objectives which have been achieved with a sound methodological design.**

**I have a few minor comments:**

Response: Thank you for your valuable comments. We have revised the manuscript to address your suggestions and provided point-by-point responses below.

**1. The authors need to explain the rationale behind the choice of the countries selected for this analysis. While it may have been done based on previous literature, were any recent data considered to justify the selection? In addition, excluding Cambodia needs a stronger justification.**

Response: We agreed with your comment. We have elaborated on this in the Methods, Line 115-121 on Page 6. Please see the changes to the manuscript below.

| **Original version** | **Revised version** |
| --- | --- |
| A decision analytic model was developed to estimate the annual economic and disease burden of snakebite in seven ASEAN countries including Malaysia, Thailand, Indonesia, Philippines, Vietnam, Lao PDR, and Myanmar. | A decision analytic model was developed to estimate the annual economic and disease burden of snakebite in seven ASEAN countries including Malaysia, Thailand, Indonesia, Philippines, Vietnam, Lao PDR, and Myanmar. These seven countries were selected based on the evidence of documented snakebite in the country and availability of local key informants to gather more insights on the situation of snakebite which were not publicly available. Brunei Darussalam and Singapore were not included because snakebite rarely occurred and/or exact data were lacking.^1^ Cambodia was not included due to lack of recently published literature on snakebite and key informants that hindered the proper estimation of the burden of snakebite in Cambodia. |

**2. The decision analytic model used in this study, assumes that complications leading to disability occur only in snakebites that require anti-venom treatment. However, there is evidence to support that both physical and psychological complications can arise in any snakebite experience independent of envenomation. The authors need to consider this phenomenon and improve the model suitably.**

Response: From the perspective of physical disability, a bite resulting in significant envenoming (i.e. indicating for antivenom) poses the greatest risk for mortality and physical disability. Thus, our model assumed that complications leading to disability occurred only in snakebite that require antivenom treatment.

We agreed that psychological complications such as PTSD could occur in any snakebite victims. Therefore, we performed sensitivity analysis that PTSD could occur in any snakebite victims and revised our findings. We have elaborated this in the Methods, Analysis, Line 209-217 on Page 9. Please see the changes to the manuscript below.

| **Original version** | **Revised version** |
| --- | --- |
| Scenario analysis was performed by incorporating post-traumatic stress disorder (PTSD) into the model as a mental disability which was modelled that PTSD would occur in 8% of the victims who survived from snakebite envenoming (**S2 Appendix** in the **supplementary material**).^63-65^ | Scenario analysis was performed by incorporating post-traumatic stress disorder (PTSD) into the model as a mental disability which estimated that PTSD would occur in 8% (95% CI; 2-18%) of the victims who survived from snakebite envenoming.^63^ PTSD could also occur following a snakebite without systemic envenoming. However, the incidence was unknown. Therefore, by applying a lower boundary level of the probability of PTSD following snakebite, it was estimated that 2% of snakebite victims without envenoming would have PTSD following snakebite. Estimation of economic burden of PTSD following snakebite is explained in **S2 Appendix** in the **supporting information**.^64-66^ |

**3. Although disability due to snakebite was confined to amputations in this study, there is evidence that many other physical complications and residual health problems result from snakebite. This phenomenon and the difficulties associated with considering them for this analysis need to be discussed.**

Response: We agreed with your comments. We have elaborated this in the Discussion, Line 372-376 on Page 16. Please see the changes to the manuscript below.

| **Original version** | **Revised version** |
| --- | --- |
| Secondly, consequences of snakebite envenoming included in the model and its sensitivity analysis were limited to death, amputation, and PTSD. Other disabilities such as blindness, malignant ulcers, and pregnancy loss were not included due to a lack of empirical evidence in ASEAN.^12^ This warranted future studies in ASEAN to evaluate all relevant consequences and disabilities of snakebite envenoming. | Secondly, consequences of snakebite included in the model and its sensitivity analysis were limited to death, amputation, and PTSD. Other disabilities such as blindness, malignant ulcers, and pregnancy loss were not included due to a lack of empirical evidence in ASEAN.^12^ This warrants future studies in ASEAN to evaluate all relevant consequences and disabilities and associated costs of snakebite to allow better estimation of burden of snakebite. |

**RESULTS**

**Reviewer #1: The results are clearly presented, both in the manuscript and in the supplemental material**

Response: Thank you for your time reviewing our manuscript.

**Reviewer #2: Due to the uncertain incidence in some countries, it is interesting to compare the data that are independent of incidence among different countries, e.g., death rate, amputation rate, average medical costs by case and average costs from productivity loss by case. This information is helpful to formulate an appropriate policy for each country.**

Response: We agreed with your comment. We have compared economic and disease burden of snakebite envenoming that are independent of incidence among different countries as shown in S3 Table in the Supplementary material.

**S3 Table.** **Estimated annual epidemiological and disease burden of snakebite envenoming per case in ASEAN countries.**

|  | Mortality rate | Amputation rate | DALYs per case | Direct costs per case, USD | Indirect costs per case, USD | Total costs per case, USD |
| --- | --- | --- | --- | --- | --- | --- |
| Malaysia | 0.004 (0.001-0.013) | - | 0.11 (0.00-0.32) | 1,736 (1,609-1,874) | 1,649 (377-4,360) | 3,386 (2,110-6,120) |
| Thailand | 0.001 (0.000-0.001) | 0.0003 (0.000-0.001) | 0.02 (0.01-0.03) | 564 (492-641) | 297 (223-377) | 861 (739-998) |
| Indonesia | 0.213 (0.107-0.420) | 0.016 (0.008-0.028) | 5.30 (2.68-10.45) | 929 (708-1,157) | 38,867 (19,690-76,612) | 39,796 (20,616-77,511) |
| Philippines | 0.313 (0.158-0.642) | 0.007 (0.004-0.008) | 7.59 (3.83-15.52 | 240 (193-292) | 46,833 (23,709-95,880) | 47,072 (23,924-96,113) |
| Vietnam | 0.040 (0.021-0.077) | - | 0.98 (0.51-1.86) | 156 (103-216) | 6,337 (3,337-12,025) | 6,493 (3,534-12,166) |
| Lao PDR | 0.332 (0.167-0.668) | 0.047 (0.007-0.115) | 8.10 (4.10-16.23) | 32 (25-40) | 26,508 (13,351-53,172) | 26,540 (13,390-53,199) |
| Myanmar | 0.132 (0.080-0.235) | - | 3.12 (1.90-5.56) | 260 (231-300) | 4,591 (2,819-8,125) | 4,851 (3,072-8,378) |
| ASEAN | 0.135 (0.089-0.234) | 0.008 (0.004-0.014) | 3.33 (2.19-5.78) | 519 (381-697) | 20,667 (12,391-38,973) | 21,186 (12,905-39,710) |

Estimates are presented as base-case estimates with their 95% credibility interval (in parentheses) based on probabilistic sensitivity analysis. Costs are presented as 2019 USD where 1 USD = 14,147.67 Indonesian Rupees = 51.80 Philippine Pesos = 23,050.24 Vietnamese Dong = 8,679.41 Lao Kip = 1,518.26 Myanmar Kyat. Abbreviations: DALYs – disability-adjusted life years; USD – US Dollar.

We have also added a paragraph regarding this comparison in the Results, Comparison of economic and disease burden per victim with snakebite envenoming across countries, Line 304-311 on Page 13-14.

***Comparison of economic and disease burden per victim with snakebite envenoming across countries***

*Economic and disease burden per victim with snakebite envenoming was compared across ASEAN countries (****S3 Table*** *in the supporting information). Mortality rate of snakebite envenoming ranged from the lowest in Thailand (0.001) to the highest in Lao PDR (0.332). Amputation rate of snakebite envenoming ranged from the lowest in Malaysia, Vietnam, and Myanmar (0.000) to the highest in Lao PDR (0.047). DALYs lost due to snakebite envenoming per victim ranged from the lowest in Thailand (0.02 DALYs per victim) to the highest in Lao PDR (8.10 DALYs per victim). Total costs of snakebite envenoming per victim ranged from the lowest in Thailand (861 USD per victim) to the highest in Philippines (47,072 USD per victim).*

**Reviewer #3: The analyses are appropriate and the results are presented comprehensively.**

Response: Thank you for your time reviewing our manuscript.

**CONCLUSIONS**

**Reviewer #1: (No Response)**

Response: Thank you for your time reviewing our manuscript.

**Reviewer #2: Line 295-296: The ASEAN burden is lower than that of Africa. Why the authors discuss that it is noticeably high?**

Response: Thank you for your comment. We apologized for our mistake in our comparison. We have compared the disease burden of snakebite across region using DALYs per 100,000 population instead. We have revised the Discussion, Line 340-344 on Page 13. Please see the changes to the manuscript below.

| **Original version** | **Revised version** |
| --- | --- |
| The estimated disease burden of snakebite of 391,979 DALYs in seven ASEAN countries was noticeably high compared to the previous estimates of 319,874 DALYs in 16 Western African countries^11^ and 1,029,209 DALYs in 41 Sub-Saharan African countries.^10^ | The estimated disease burden of snakebite of 391,979 DALYs in seven ASEAN countries (61 DALYs per 100,000 population) was low compared to the previous estimates of 319,874 DALYs in 16 Western African countries (approximately 93 DALYs per 100,000 population)^11^ and 1,029,209 DALYs in 41 Sub-Saharan African countries (approximately 120 DALYs per 100,000 population).^10^ This could be partly explained by the differences in the incidence and mortality of snakebite and access to antivenom treatment. |

**Reviewer #3: The conclusions are appropriate.**

**However, a stronger conclusion towards prevention, improving access to anti-venom and better management of snakebite will help to inform policy in the ASEAN region.**

Response: We agreed with your comment. We have elaborated more in the Conclusion, Line 389-399 on Page 16-17. Please see the changes to the manuscript below.

| **Original version** | **Revised version** |
| --- | --- |
| Our estimates highlighted the high economic and disease burden of snakebite in ASEAN despite the availability of domestically produced antivenoms. Almost all of the estimated economic and disease burdens were attributed to premature deaths from snakebite envenoming which suggested that the remarkably high burden of snakebite could be averted, especially in countries where large proportions of victims who needed antivenom were not treated with geographically appropriate antivenoms. | Annual production of 290,000 vials of antivenom in ASEAN were given to only 31% of victims who were indicated for antivenom treatment. Our estimates highlighted the high economic and disease burden of snakebite in ASEAN despite the availability of domestically produced antivenoms. Almost all of the estimated economic and disease burdens were attributed to premature deaths from snakebite envenoming which suggested that the remarkably high burden of snakebite could be averted, especially in countries where large proportions of victims who needed antivenom were not treated with geographically appropriate antivenoms. Strategies should be developed with the goal to improve health outcomes of snakebite victims. However, strategies used to achieve this goal are likely to be complex and different across countries depending on each country’s context and situation such as accurate informatics, rigorous regulations of antivenoms, efficient supply chain, rational use of antivenoms, appropriate treatment seeking behaviors, and good governance to support a strong healthcare system. |

**Editorial and Data Presentation Modifications?**

**Reviewer #1: This important manuscript address a greatly under-researched and greatly-needed domain in the field of snakebite and I congratulate the authors for their efforts in acquiring data from very diverse sources.**

Response: Thank you for your time reviewing our manuscript.

**The manuscript is well structured but would benefit from extensive editing on English phrasing and spelling.**

Response: Thank you for your comment. Our manuscript has been proofread by native English speaker.

**Line 106 - there are so few papers examining the HE costs of snakebite that I was surprised to see that the PLOS paper on the HE burden of snakebite in Burkina Faso was not included - it should be (Ahmed S, Koudou GB, Bagot M, Drabo F, Bougma WR, Pulford C, Bockarie M, Harrison RA. Health and economic burden estimates of snakebite management upon health facilities in three regions of southern Burkina Faso. PLoS Negl Trop Dis. 2021 Jun 21;15(6)). The absence of this paper suggests that the authors need to run an updated extensive review of the global literature for snakebite HE studies.**

Response: Thank you for your comment. We have added the suggested article to our introduction.

**If the authors are sufficiently confident in their HE data and analysis, I think the title should be revised to increase its impact and likelihood of being read by Public Health decision makers - perhaps something like 'Health economic analysis estimates that the 391,979 DALY annual disease burden of snake envenoming in the ASEAN region costs USD 2.5 billion'.**

Response: Thank you for your suggestion. However, we decide not to revise the title.

**I think the manuscript would benefit from an expansion of the overly brief discussion. This could include implications to WHO and each ASEAN country to meet the target of halving snakebite mortality and morbidity by 2030 (more primary research delivering data with fewer assumptions; cost benefits of managing snakebite better; funding implications - by Governments and WHO to meet this target; etc)**

Response: We agreed with your comment. We have elaborated more in the Discussion, Line 357-367 on Page 15. Please see the changes to the manuscript below.

| **Original version** | **Revised version** |
| --- | --- |
| Treating all snakebite victims who need antivenoms in these countries would save their lives which would result in tremendous decrease in the burden of snakebite in ASEAN. However, increasing access to antivenom was not about only producing antivenoms but the whole surrounding supporting and management system especially information system to inform decision making and logistics to efficiently deliver antivenoms even to the farthest healthcare facilities. | Treating all snakebite victims who need antivenoms in these countries would save their lives which would result in a tremendous decrease in the burden of snakebite in ASEAN. However, increasing access to antivenom was not only about producing antivenoms but the whole surrounding supporting and management system especially the information system to inform decision making and logistics to efficiently deliver antivenoms even to the farthest healthcare facilities. We previously assessed the situation of snakebite in ASEAN and provided the potential opportunities to improve situation of snakebite in ASEAN to meet the WHO’s target of halving snakebite mortality and morbidity by 2030. These potential opportunities included accurate estimation of antivenom demand, rigorous regulations of antivenom, strengthening the supply chain system, raising public awareness about the importance of treating snakebite envenoming by healthcare professionals, strengthening the health system to ensure appropriate snakebite management and rational use of antivenoms, and expanding collaboration of local and international stakeholders and funders.^5^ |

**The conclusion would benefit by a very clear statement that the annual delivery of 290,000 vials of antivenom (apprx 42,000 treatments) is less than half that needed to treat the 117,575 victims - and describe the medical, societal, USD and GDP cost benefits of meeting the cost of delivery this expanded volume of antivenom.**

Response: We agreed with your comment. We have elaborated more in the Conclusion, Line 389-399 on Page 16-17. Please see the changes to the manuscript below.

| **Original version** | **Revised version** |
| --- | --- |
| Our estimates highlighted the high economic and disease burden of snakebite in ASEAN despite the availability of domestically produced antivenoms. Almost all of the estimated economic and disease burdens were attributed to premature deaths from snakebite envenoming which suggested that the remarkably high burden of snakebite could be averted, especially in countries where large proportions of victims who needed antivenom were not treated with geographically appropriate antivenoms. | Annual production of 290,000 vials of antivenom in ASEAN were given to only 31% of victims who were indicated for antivenom treatment. Our estimates highlighted the high economic and disease burden of snakebite in ASEAN despite the availability of domestically produced antivenoms. Almost all of the estimated economic and disease burdens were attributed to premature deaths from snakebite envenoming which suggested that the remarkably high burden of snakebite could be averted, especially in countries where large proportions of victims who needed antivenom were not treated with geographically appropriate antivenoms. Strategies should be developed with the goal to improve health outcomes of snakebite victims. However, strategies used to achieve this goal are likely to be complex and different across countries depending on each country’s context and situation such as accurate informatics, rigorous regulations of antivenoms, efficient supply chain, rational use of antivenoms, appropriate treatment seeking behaviors, and good governance to support a strong healthcare system. |

**The authors could also make the important point, perhaps after the 'limitations' section) that funding of primary research would deliver much needed snakebite HE data that would reduce the data assumptions (that had to be made to complete this analysis) and yield accurate HE data for ASEAN countries, and WHO, to guide their decision making.**

Response: We agreed with your comment. We have elaborated more in the Discussion, Line 383-386 on Page 16. Please see the changes to the manuscript below.

| **Original version** | **Revised version** |
| --- | --- |
| We emphasized the importance of conducting a comprehensive data collection on epidemiological and economic burden of snakebite to eventually reveal the true burden of snakebite in ASEAN. | We emphasized the importance of funding research to perform a comprehensive data collection on epidemiological and economic burden of snakebite to eventually reveal the true burden of snakebite in ASEAN. These data will yield more accurate information on burden of snakebite to guide decision making in not only the ASEAN but also the WHO to develop global strategies to tackle the problem of snakebite. |

**Reviewer #2: (No Response)**

Response: Thank you for your time reviewing our manuscript.

**Reviewer #3: Minor Revision**

Response: Thank you for your time reviewing our manuscript.

**SUMMARY AND GENERAL COMMENTS**

**Reviewer #1: This is an important paper that adds valuable new information on the disease and health economic burden caused by snakebite. It could, but shouldn't, be criticised for the many data assumptions that were needed to populate their analytical tree - because the data was the best available and the results and public health implications therefrom are very important.**

Response: Thank you for your time reviewing our manuscript.

**Reviewer #2: The knowledge of the disease burden in this area will be very helpful for the policy makers.**

Response: Thank you for your time reviewing our manuscript.

**1. The incidence and burden are markedly heterogeneous among different countries suggesting that the policy changes should be country-specific.**

Response: We agreed with your comment. We have elaborated more in the Discussion, Line 357-367 on Page 15. Please see the changes to the manuscript below.

| **Original version** | **Revised version** |
| --- | --- |
| Treating all snakebite victims who need antivenoms in these countries would save their lives which would result in tremendous decrease in the burden of snakebite in ASEAN. However, increasing access to antivenom was not about only producing antivenoms but the whole surrounding supporting and management system especially information system to inform decision making and logistics to efficiently deliver antivenoms even to the farthest healthcare facilities. | Treating all snakebite victims who need antivenoms in these countries would save their lives which would result in a tremendous decrease in the burden of snakebite in ASEAN. However, increasing access to antivenom was not only about producing antivenoms but the whole surrounding supporting and management system especially the information system to inform decision making and logistics to efficiently deliver antivenoms even to the farthest healthcare facilities. We previously assessed the situation of snakebite in ASEAN and provided the potential opportunities to improve situation of snakebite in ASEAN to meet the WHO’s target of halving snakebite mortality and morbidity by 2030. These potential opportunities included accurate estimation of antivenom demand, rigorous regulations of antivenom, strengthening the supply chain system, raising public awareness about the importance of treating snakebite envenoming by healthcare professionals, strengthening the health system to ensure appropriate snakebite management and rational use of antivenoms, and expanding collaboration of local and international stakeholders and funders.^5^ |

**2. The burden is largely contributed by the countries where the snakebite incidences were very high and estimated by local experts. This limitation should be addressed.**

Response: We agreed with your comment. We have elaborated more in the Discussion, Line 377-381 on Page 16. Please see the changes to the manuscript below.

| **Original version** | **Revised version** |
| --- | --- |
| Lastly, there was no nation-wide community and hospital study to comprehensively collect the number of snakebite victims in some of the included countries. Hence, input parameters must be estimated based on non-national studies, unpublished local data, and expert opinions, resulting in a wide range of the estimated economic and disease burden of snakebite in ASEAN. | Lastly, there was no nation-wide community and hospital study to comprehensively collect the number of snakebite victims in some of the included countries. Hence, input parameters must be estimated based on non-national studies, unpublished local data, and expert opinions, resulting in a wide range of the estimated economic and disease burden of snakebite in ASEAN. This is especially relevant in Lao PDR and Indonesia where snakebite incidences were very high and estimated by local experts. |

**Reviewer #3: Overall this is a very good attempt at quantifying the burden of snakebite in the ASEAN region and will add significantly to the literature on snakebite.**

Response: Thank you for your time reviewing our manuscript.
